# Ab Initio Molecular Dynamics Study of Trivalent Rare Earth Rich Borate Glasses: Structural Insights and Formation Mechanisms

**DOI:** 10.1021/acs.jpcb.4c05039

**Published:** 2024-11-11

**Authors:** Takahiro Ohkubo, Shunta Sasaki, Atsunobu Masuno, Eiji Tsuchida

**Affiliations:** † Graduate School of Engineering, Chiba University, 1-33 Yayoi-cho Inage-ku, Chiba 263-8522, Japan; ‡ Department of Material Chemistry, Graduate School of Engineering, Kyoto University, Kyotodaigaku-Katsura, Nishikyo-ku, Kyoto 615-8520, Japan; ¶ National Institute of Advanced Industrial Science and Technology (AIST), Tsukuba Central 2, Umezono 1-1-1, Tsukuba 305-8568, Ibaraki, Japan

## Abstract

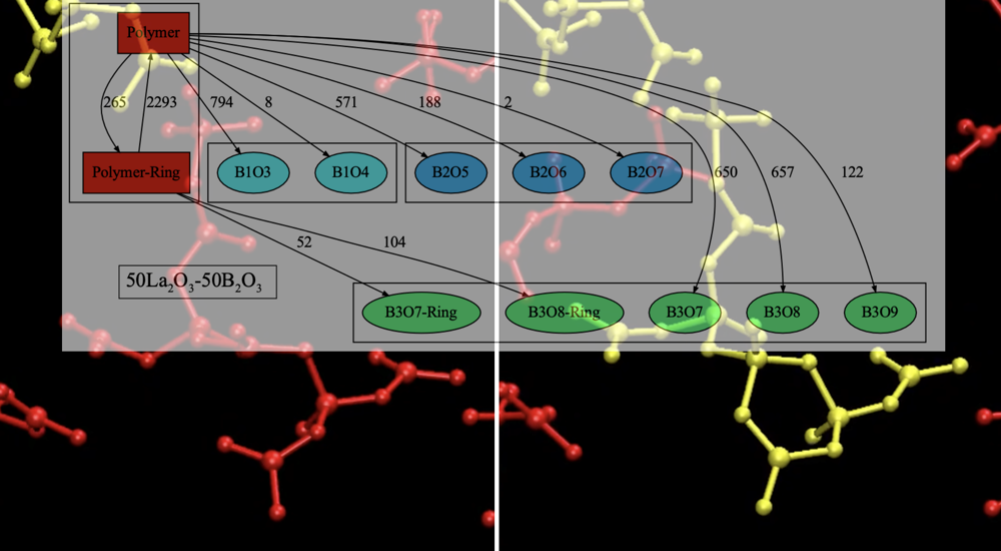

In this work, trivalent rare earth (RE)-rich borate glasses (30 La_2_O_3_-70 B_2_O_3_, 50 La_2_O_3_-50 B_2_O_3_, 60 La_2_O_3_-40 B_2_O_3_, and 50 Y_2_O_3_-50 B_2_O_3_) were modeled using ab initio molecular dynamics (AIMD) simulations through the melt-quenching route. It was found that the AIMD-derived structures reproduced the experimental structure factors and ^11^B solid-state nuclear magnetic resonance data. Isolated borate units (monomers, dimers, and trimers) terminated with nonbridging oxygen were found in the structures. Polymer units containing four or more boron atoms were identified with and without three-membered boron rings (3-rings). Increasing the proportion of La_2_O_3_ in La_2_O_3_–B_2_O_3_ glasses resulted in an increased number of isolated units, indicating that La^3+^ acts as a network modifier, breaking the borate glass network. The formation of these units via the melt-quenching process was detected by labeling boron species at each AIMD step from 1500 to 300 K. Representation with transition matrices clarified the specific reaction routes, leading to the formation of isolated boron units in solid glass. A key finding is the stabilization of polymer units involving 3-ring formation. The formation of isolated units is achieved through the reaction of polymers without 3-rings. The RE coordination structure was thoroughly analyzed from the perspective of shape and symmetry. Reference structures derived from the solution of the Thomson problem were compared to the AIMD-derived coordination structures and crystalline LaBO_3_ and YBO_3_. The results highlight the specificity of the Y coordination structure with 3-rings in YBO_3_, which is not observed in RE borate glasses. The analytical approaches and interpretations used in this study provide insights into the diverse coordination structures of glasses containing heavy elements other than REs.

## Introduction

Glasses containing trivalent rare earths (REs) exhibit unique luminescence and mechanical properties^1−8^; however, compared to common silicate glass systems containing light-element alkalis and alkaline earths, there is a lack of understanding of their structures. In the 15 lanthanide elements (La through Lu), the seven 4*f* orbitals are filled sequentially by 14 electrons, and all have 5*s*^2^ 5*p*^6^ electron configurations in their outermost shells. Because of this common electron configuration, the chemical properties of each RE element are similar.

One example of glasses containing a RE is the lanthanum borate system, which exhibits a high refractive index due to the presence of heavy RE ions.^9−14^ The large number of electrons in RE elements play a key role in increasing the refractive index of optical glasses; furthermore, RE-doped optical-amplification fibers and low-loss optical fibers are currently being researched for new applications in advanced optical communication.^13,15−18^ Trivalent RE elements can also be generated as fission products of nuclear fuel, and they are found in spent nuclear fuel.^19,20^ Because nuclear waste is mixed and solidified into borosilicate glass for its final disposal, it is essential to understand the nature of borate glass containing trivalent REs to gain a fundamental understanding of nuclear-waste immobilization.^19,21−23^

Binary *x* La_2_O_3_–(1 – *x*) B_2_O_3_ glass systems have been studied in a wide composition range. Glasses with *x* = 0.25–0.28 can form homogeneously without liquid–liquid phase separation, and they show high refractive indices (*n* = 1.70–1.77).^9^ Glasses with RE-rich compositions represent a new group of materials, and they have been extensively studied in recent years^10,24−26^; these new materials are known to exhibit characteristic optical properties related to their peculiar structural features.^10^

It has been suggested that the introduction of excess trivalent REs in borate glasses can produce abundant nonbridging oxygen for charge compensation, resulting in isolated BO_3_^3–^ units, as revealed by solid-state ^11^B and ^17^O nuclear magnetic resonance (NMR) measurements.^10,26^ Such an isolated BO_3_^3–^ structure formed solely from nonbridging oxygen is also found in the LaBO_3_ crystal, as shown in Figure S1, in which the charge of La^3+^ is balanced by three nonbridging oxygens.^27^ Interestingly, the borate structure in YBO_3_ with the same trivalent RE has a ring with three BO_4_^–^ units in contrast to the isolated BO_3_ units in LaBO_3_,^28^ as shown in Figure S1. In a previous report,^29^ we explained that this structural change in borate units for YBO_3_ and LaBO_3_ was the result of a difference in the ionicity of Y^3+^ and La^3+^.

The importance of intermediate structures for understanding the overall structures of borate glasses has been recognized in both experimental and theoretical studies.^30−34^ It is expected that intermediate-range-ordered structures are caused by the rearrangement of isolated or small-chain units with BO_4_ and BO_3_ around the glass-transition temperature. A variety of intermediate-range structures have been found in crystals and borate units involving three-membered boron rings (3-rings).^35^

The final structures obtained via melt-quenching synthesis of borate glasses is the result of an equilibrium obtained through a process of repeated bond formation and cleavage in the molten state. Experimental studies of structural changes from glass melts to solidification have been limited due to the limitations of experimental setups. High-temperature Raman-scattering experiments with pure borate and binary sodium borates have been performed over the range from 1200 °C to room temperature.^36−41^ The boroxol rings in pure borate glass tend to be destroyed by thermal excitation in the molten state^37^; however, equilibrium reactions between boroxol rings and nonring borate units in the molten state have been proposed for binary sodium borate melts.^39−41^ The conversion reaction from BO_4_ to BO_3_ and nonbridging oxygen has also been observed in glasses above 20 mol % sodium content. These reactions involving the formation and breaking of bonds in the molten state are key to understanding the origin of the structure of the resultant borate glasses.

Computational experiments using molecular dynamics (MD) calculations are an important approach to accessing the structures of molten and solidified glasses. Potential functions, which are explicit functional forms for distances and angles, have been developed to reproduce experimental data obtained using borate glasses to create realistic glass models.^42−44^ This type of MD calculations is sometimes referred to as classical MD (CMD) to contrast it with ab initio calculations. Although a certain amount of success in understanding the structures of glasses has been achieved with CMD, it is known that the unique superstructures found in borate glasses, such as boroxol rings, are not reproduced well using this approach.^38,45−48^

Ab initio MD (AIMD) calculations based on density functional theory (DFT) can be used to analyze the structures of glasses at the expense of high computational cost. It has been reported that the dynamics and structures of borate glasses obtained using AIMD can reproduce experimental data; because the boroxol ring structure found in borate glasses is well represented in AIMD,^45,49^ it is possible to analyze the role of intermediate structures in borate glass networks.

The purpose of this study was to clarify the formation mechanisms of unique borate units in trivalent RE (La and Y) borate glasses. A series of simulations from melts to solidified glasses were performed using AIMD, and the resulting glass structures were compared to experimental data. The monitoring of the solidification process of the glass melt allowed for the examination of the reaction mechanisms of isolated or chained borate structures, including the 3-ring structure. Examining the structural transitions of boron species with a time step of the order of femtoseconds can shed light on the reaction mechanisms of diverse boron structural units, which are difficult to access through CMD and experimental approaches. We investigated the reaction mechanisms in the process from glass melt to solidification using a representation based on the reaction transition matrix. A comprehensive analysis of the coordination structure of REs in the borate glass was also performed based on coordination shape and symmetry to highlight the differences in RE types.

## Methods

### AIMD Simulations

Four RE borate glass compositions containing La and Y were selected for AIMD calculations; these are listed in Table 1, along with their labels and experimentally measured densities.^29^ These glass compositions were taken from our previous experimental study.^29^ In each case, the simulation box contained 500 atoms in accordance with the compositions. The dimensions of the cubic box were set based on the experimentally measured density.

**Table 1:** Composition and Density (ρ) in g/cm^3^ of the Glass Systems for AIMD Calculations^a^

label	composition	ρ	*L*	*N*	La	B	O	Y
a-La30	30 La_2_O_3_-70 B_2_O_3_	4.37	17.72	500	60	140	300	
a-La50	50 La_2_O_3_-50 B_2_O_3_	5.46	18.18	500	100	100	300	
a-La60	60 La_2_O_3_-40 B_2_O_3_	5.52	18.87	500	120	80	300	
a-Y50	50 Y_2_O_3_-50 B_2_O_3_	4.57	17.51	500		100	300	100

a The side length of the cubic cell (*L*) in Å, the total number of atoms (*N*), and the number of atoms of each element are also listed.

The initial structure was created by randomly placing atoms in the cell. The AIMD calculations were performed using our DFT code, the Finite-Element-Method-based Total Energy Calculation Kit (FEMTECK), which enables high-efficiency DFT computations of amorphous materials on massively parallel supercomputers.^50−52^ All production runs were performed with an average cutoff energy of 75–85 Ry. Norm-conserving pseudopotentials were used to represent the ionic cores.^53,54^ Realistic atomic and electronic structures of oxide glasses, including boron-ring structures, have been successfully constructed via a melt-quenching route using FEMTECK.^45,48^ In each case, the initial structure was equilibrated at 4000 K for 80 ps, and the molten glass was subsequently cooled to 300 K at a cooling rate of 20 K/ps. Once the system temperature had reached 300 K, a production run for 100 ps was performed to sample the atomic structure of the solid glass.

All AIMD simulations were conducted in the canonical (NVT) ensemble with a Berendsen thermostat^55^ and a 1.0 fs time step. The B–O bonds were determined based on the interatomic cutoff distance, and the borate structural units were identified at each step. Structural optimizations of the AIMD-derived glass structures obtained at the last time step were performed to investigate the atomic and electronic structures of the RE coordination sphere; the atomic positions were optimized to achieve a force convergence of 10^–3^ eV/Å for the force components acting on each atom; structural optimizations for the crystalline LaBO_3_ and YBO_3_ were also performed to obtain their electron densities for comparison purposes.

### High-Energy X-ray Diffraction

The samples from our previous study were used for high-energy X-ray diffraction (HEXRD) measurements.^29^ Each of these samples was prepared using a levitation technique combined with CO_2_ laser heating. These HEXRD data were collected at SPring-8 (BL04B2 beamline) using a two-axis diffractometer and four CdTe detectors and three Ge detectors. The powdered samples were filled into capillary tubes and placed in a vacuum chamber, and the measurements were performed at room temperature. To obtain the total structure factor *S*(*Q*), the diffraction data were corrected for absorption, background, and polarization, and they were normalized.

### Solid-State NMR

^11^B magic-angle spinning (MAS) NMR measurements of the a-La30 glass, which were not undertaken in our previous study,^29^ were collected for comparison with the AIMD results. The ^11^B MAS NMR experiments were conducted with a pulse width of 0.33 μ s and a 15-kHz spinning speed. The recycle delays for complete relaxation and signal accumulation were 30 s and 64, respectively. The reference for the chemical shifts was a 1.0-M aqueous solution of H_3_BO_3_ at 19.5 ppm. Spectral simulations of the ^11^B MAS NMR spectra were conducted using an in-house program incorporating the mrsimulator Python package.^56,57^

## Results and Discussion

### Glass Structures

**Figure 1:**
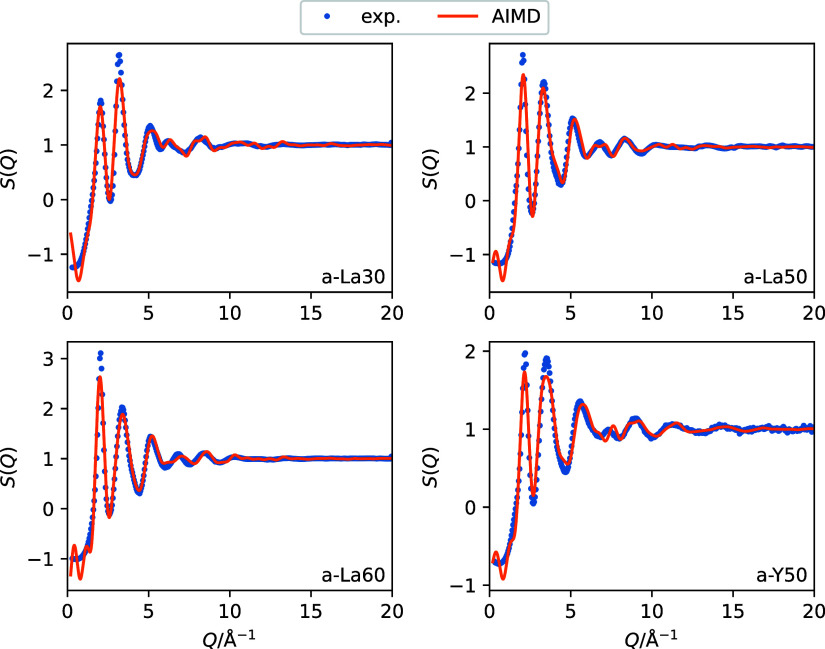
Experimental and AIMD-calculated *S*(*Q*) values for the four glass systems. The calculated *S*(*Q*) values were obtained from time-averaged analysis of the AIMD-derived glass structures.

The validity of the structures generated by the AIMD calculations was checked by comparing the structure factors *S*(*Q*) calculated from AIMD-derived structures with the experimental data as shown in Figure 1. Herein, *S*(*Q*) is defined as follows:
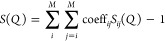
1
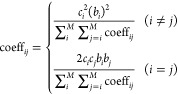
2where *Q* is the scattering vector, *c*_*i*_ and *c*_*j*_ are the atomic fractions in the glass, *b*_*i*_ and *b*_*j*_ are the X-ray scattering coefficient, *i* and *j* correspond to atomic types with *M* = 3 (La, B, and O). The partial scattering structure factor, *S*_*ij*_(*Q*), can be calculated from the partial radial distribution function, *g*_*ij*_(*r*). Details of this procedure are available in other studies.^58−60^

The equilibrated structures at 300 K were sampled every 100 fs and a time-averaged *S*(*Q*) was calculated. A peak near 2.0 Å^–1^ and a subsequent peak near 3.2 Å^–1^ were observed in the four glass systems. The intensities and positions of these peaks were dependent on the composition. The *S*(*Q*) values calculated from the AIMD-derived structures showed good agreement with experimental data in terms of intensity and phase. Furthermore, structural features were found to be well reproduced by the AIMD-based procedures used in this study.

**Figure 2:**
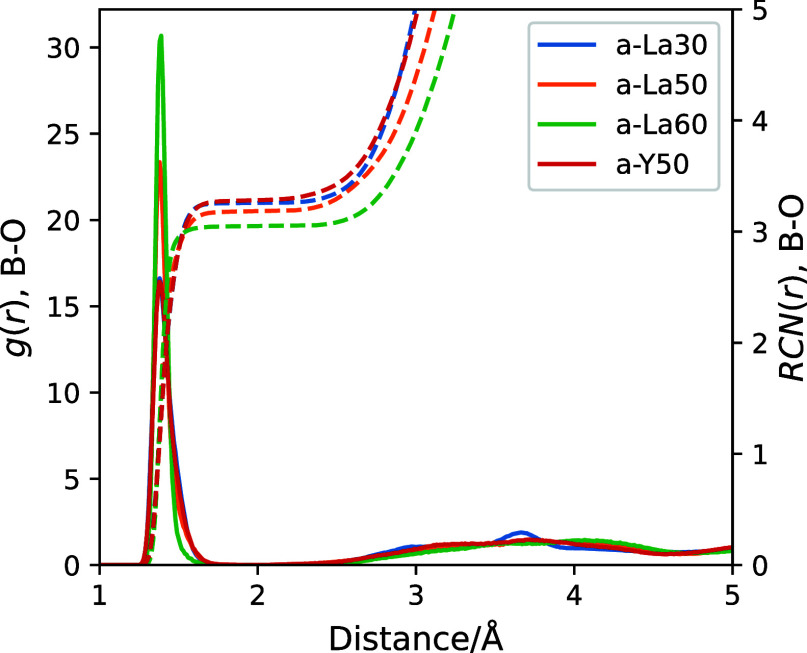
Radial distribution functions [*g*(*r*), solid lines] and running coordination numbers [RCN(*r*), dashed lines] of B–O estimated from AIMD-derived RE glass structures at 300 K.

The radial distribution functions *g*(*r*) and running coordination numbers RCN(*r*) of the B–O pairs were calculated from the structures obtained at 300 K as shown in Figure 2. These functions are defined as follows:
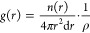
3
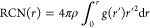
4where *n*(*r*) is the number of O atoms within a shell volume of thickness d*r* at distance *r*, and ρ is the number density of O atoms.

The maximum of the first peak was found at 1.38 Å for the four glass systems. Three- and four-coordinated B (B^3^ and B^4^) were identified by counting coordination numbers within a cutoff distance of 2.0 Å. The mean B^4^–O and B^3^–O bond lengths were individually calculated, and they are listed in Table 2. The experimental B^3^–O and B^4^–O bond distances reported in alkali borate glasses are 1.37 and 1.47 Å, respectively.^61,62^ Both the B^3^–O and B^4^–O bond distances obtained from the AIMD-derived structures are slightly longer than those reported for other borate glasses. This trend can be attributed to the nature of trivalent RE borate glass rather than to errors in the AIMD results; indeed, the mean B^3^–O and B^4^–O distances in LaBO_3_ and YBO_3_ crystals have been measured as 1.435 and 1.500 Å,^27,28^ longer than those in alkali borate crystals.

**Table 2:** Bond Lengths of B^3^–O and B^4^–O Pairs for RE Glasses^a^

label	B^3^–O	B^4^–O
a-La30	1.387 (0.044)	1.494 (0.063)
a-La50	1.388 (0.038)	1.503 (0.078)
a-La60	1.392 (0.035)	1.511 (0.080)
a-Y50	1.387 (0.043)	1.496 (0.077)

a Values in parentheses indicate standard deviations.

Experimental and simulated ^11^B MAS NMR spectra for 30 La_2_O_3_-70 B_2_O_3_ glass are shown in Figure S2, and the corresponding ^11^B NMR parameters are listed in Table S1. Two B^3^ species (ring and nonring) and one B^4^ species were included in the simulations of the experimental ^11^B MAS NMR spectra considering second-order quadrupolar interactions under an infinitely fast spinning speed. In addition to the a-La30 ^11^B MAS NMR results, the percentages of B^4^ obtained from our previous NMR spectra^29^ were compared to those in the glass structures obtained from the AIMD calculations.

**Figure 3:**
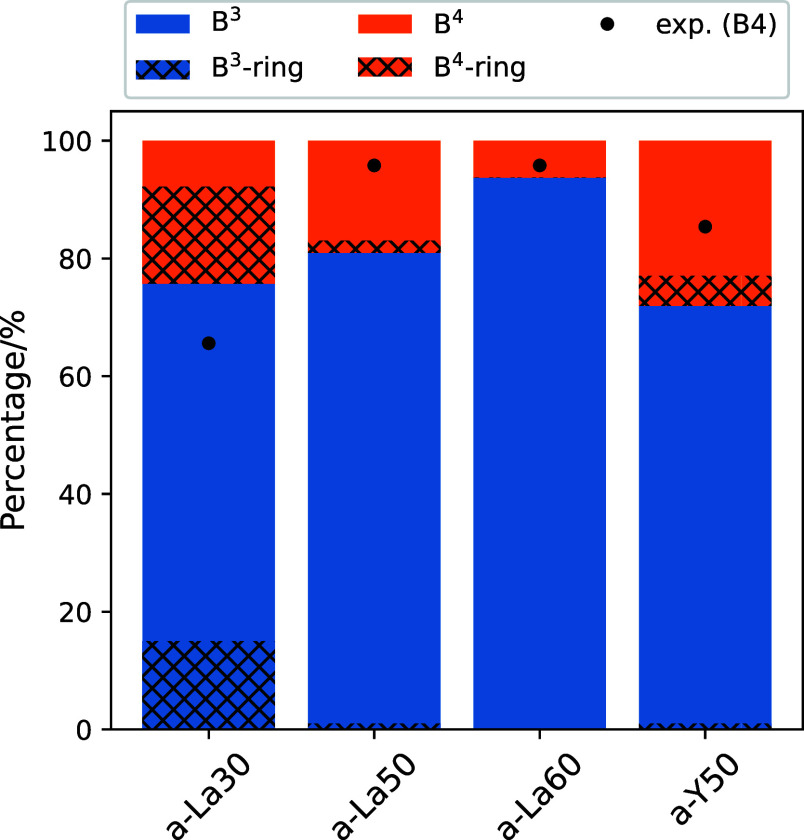
Percentages of boron species identified by coordination number in the AIMD-derived structures. The heights of the hatched areas correspond to the population of B participating in 3-rings. The black circles indicate experimental results derived from ^11^B MAS NMR spectra.

The percentages of boron differentiated by coordination number are displayed in Figure 3 along with the B^4^ percentages obtained from the experimental ^11^B MAS NMR spectra. The B^3^ and B^4^ were further classified according to whether or not they constitute a 3-ring. The heights of the hatched bars in Figure 3 indicate the percentages of boron participating in 3-rings with B^3^ and B^4^. In AIMD-derived glass models, where the cell size is limited by computational costs, the B^4^ percentage fluctuates due to the small number of boron atoms present in the cell. This variation has been studied in CMD simulations of lithium borosilicate glasses containing approximately 100 boron atoms.^45^ The standard deviation of the B^4^ percentage was found to be 4%. Therefore, the B^4^ percentage reported in Figure 3 should be considered subject to a similar level of variation.

**Figure 4:**
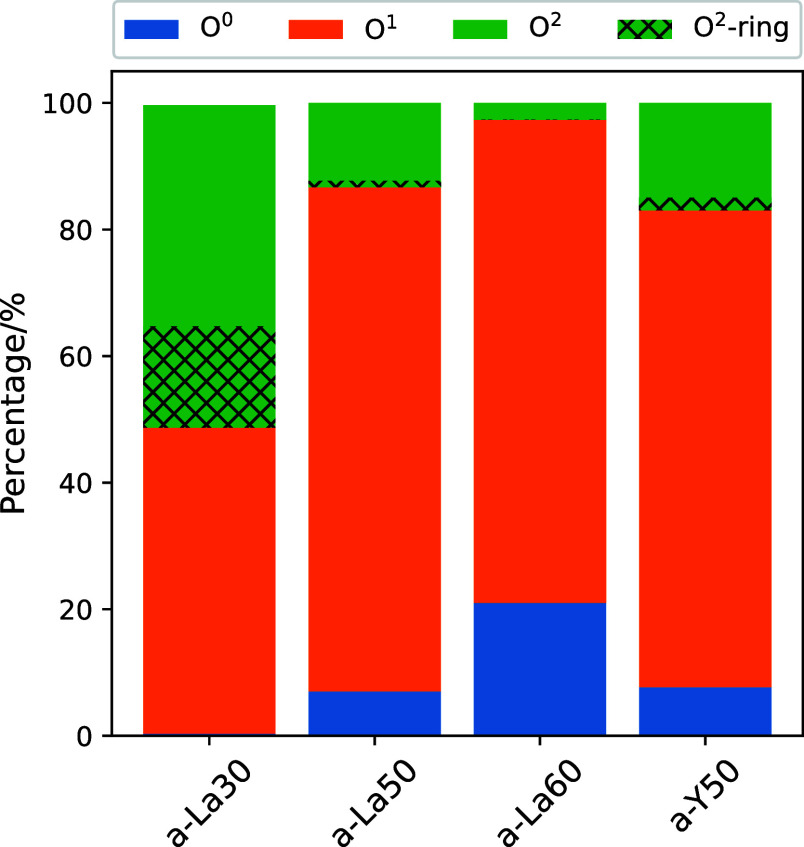
Percentages of oxygen species identified by the number of bonded boron atoms. The heights of the hatched regions correspond to the percentages of oxygen in 3-rings.

The percentages of B^4^ obtained from the ^11^B MAS NMR experiments for all compositions showed a discrepancy of about 10% from the AIMD-derived structures. Considering the broadened line shape resulting from structural dispersion and the simulation model for quadrupolar nuclei, the percentages of B^4^ determined from the experiments may need to account for an error of about 10%. The discrepancy could possibly be caused by composition changes during the high-temperature synthesis. In fact, the percentages of B^4^ in La_2_O_3_–B_2_O_3_ glass reported by Masuno et al.^10^ and Swanson et al.^26^ show a discrepancy of about 5% despite having the same composition. Furthermore, the glass structures derived from the AIMD simulations were generated at extremely high cooling rates (20 K/ps) compared to the experimental conditions (∼100 K/s). This may result in there being insufficient conversion from B^4^ to B^3^ and nonbridging O. However, the AIMD calculations can reproduce the decrease in B^4^ with increasing La_2_O_3_ in La_2_O_3_–B_2_O_3_ glass. Furthermore, the percentage of B^4^ in a-La50 is higher in a-Y50 glass than that of a-La50, which have the same RE contents. This trend found in AIMD-derived structures is consistent with the results from ^11^B MAS NMR experiments.

Oxygen species were also identified by the number of bonded boron atoms. The a-La30 glass was composed of O^1^ and O^2^, including O^2^ in 3-rings (O^2^-rings), where the superscript number indicates the number of bonded boron atoms. The generation of O^0^ without boron bonds was found in the glasses with higher La_2_O_3_ contents. It should be noted that O^0^ is coordinated to trivalent RE and not to isolated atoms. The O^1^ content in all the glasses except for a-La30 was about the same. Excess RE in the glass leads to an increase in O^0^ and a decrease in O^2^, suggesting that O^1^ species terminating the boron unit are saturated at RE contents above 50% La_2_O_3_.

**Figure 5:**
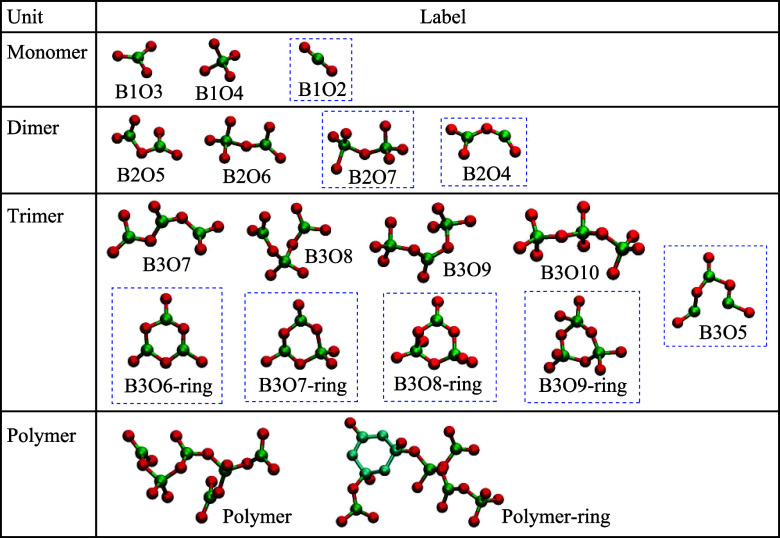
All identified boron units found in the AIMD simulations from the molten to solidified state (1500–300 K). The red and green balls correspond to oxygen and boron atoms. Atoms in 3-ring units in polymers are drawn in cyan for ease of recognition. All units are terminated with O^1^. The numbers of boron and oxygen atoms in monomer, dimer, and trimer units are represented as digits following the elemental symbol in the label. All units including four or more boron atoms are classified as polymers. The labels of units containing 3-rings have “-ring” appended.

The boron units were classified into *n*-mer units to analyze the isolated or chain structure, where *n* is the number of boron atoms in the units. The end of the boron units are terminated with O^1^. All detected monomer, dimer, and trimer structures corresponding to *n* = 1, 2, and 3 in the four glass systems for the temperature range from 1500 to 300 K are displayed in Figure 5. The number of boron and oxygen atoms in these units are represented as digits following the elemental symbol in the label. The units with *n* > 3 are grouped together as a “polymers.” If a 3-ring structure is included in the units, the extension “-ring” is attached to the label. The units surrounded by blue dashed lines in Figure 5 are structures that were absent from the solid glasses at 300 K and were thus detected only in the molten state. It should be noted that no other boron units than those presented in Figure 5 were found during the AIMD simulations in this study. For instance, no isolated diborate unit consisting of two 3-rings was detected in either the molten or solid glass; such isolated units involving double 3-rings may be absent from RE-rich glass due to significant structural modification by the REs.

**Figure 6:**
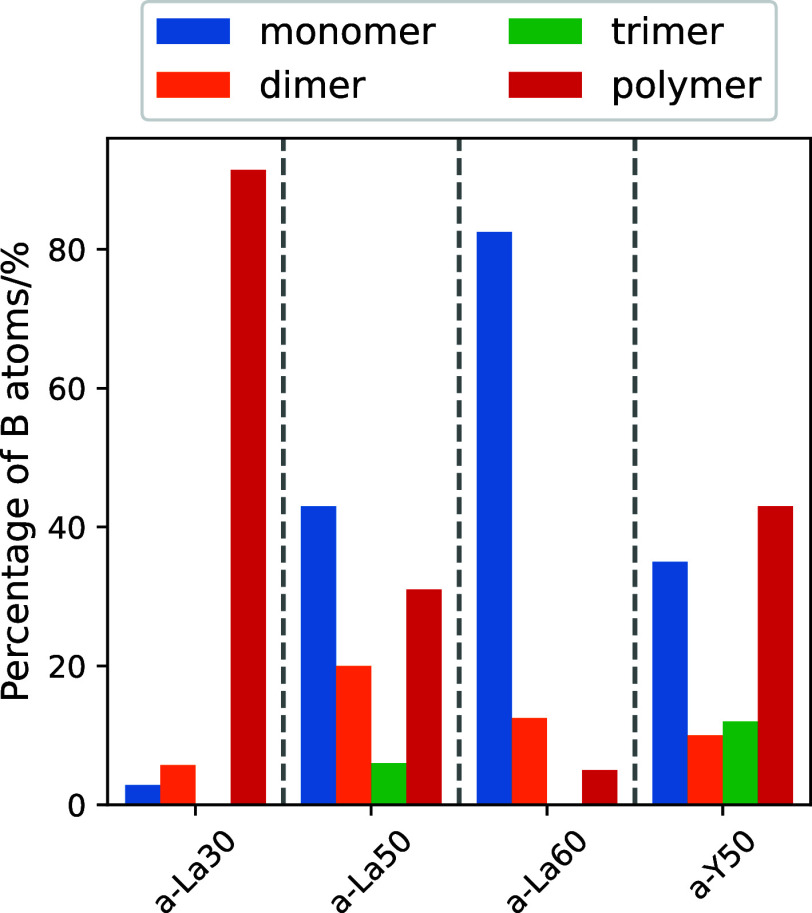
Numbers of boron atoms belonging to monomer, dimer, trimer, and polymer units in the four glass systems at 300 K.

The percentages of boron belonging to each structural unit (monomer, dimer, trimer, and polymer) in the four glass systems are shown in Figure 6. Boron atoms in polymer units are the majority in the a-La30 glass, and the percentage of boron atoms in monomer and dimer units increased with increasing RE content. This trend implies that RE atoms in the glass play the role of network modifiers to break the borate glass network. The percentage of boron in monomer or dimer form in the a-La50 glass was found to be greater than that in the a-Y50 glass. The degree to which La^3+^ induces structural modification was found to be higher than that caused by Y^3+^. Changes in the glass structure depending on the trivalent ionic species (La or Y) will be further discussed in the analysis of the atomic coordination and electronic structure of these glasses in the subsection “RE Coordination Structure.”

To elucidate the origin of the variety of boron units in the solid glass, a reaction analysis during the quenching process was undertaken with a view to providing deep insights into the formation mechanisms of these diverse boron structural units.

### Formation Mechanisms

**Figure 7:**
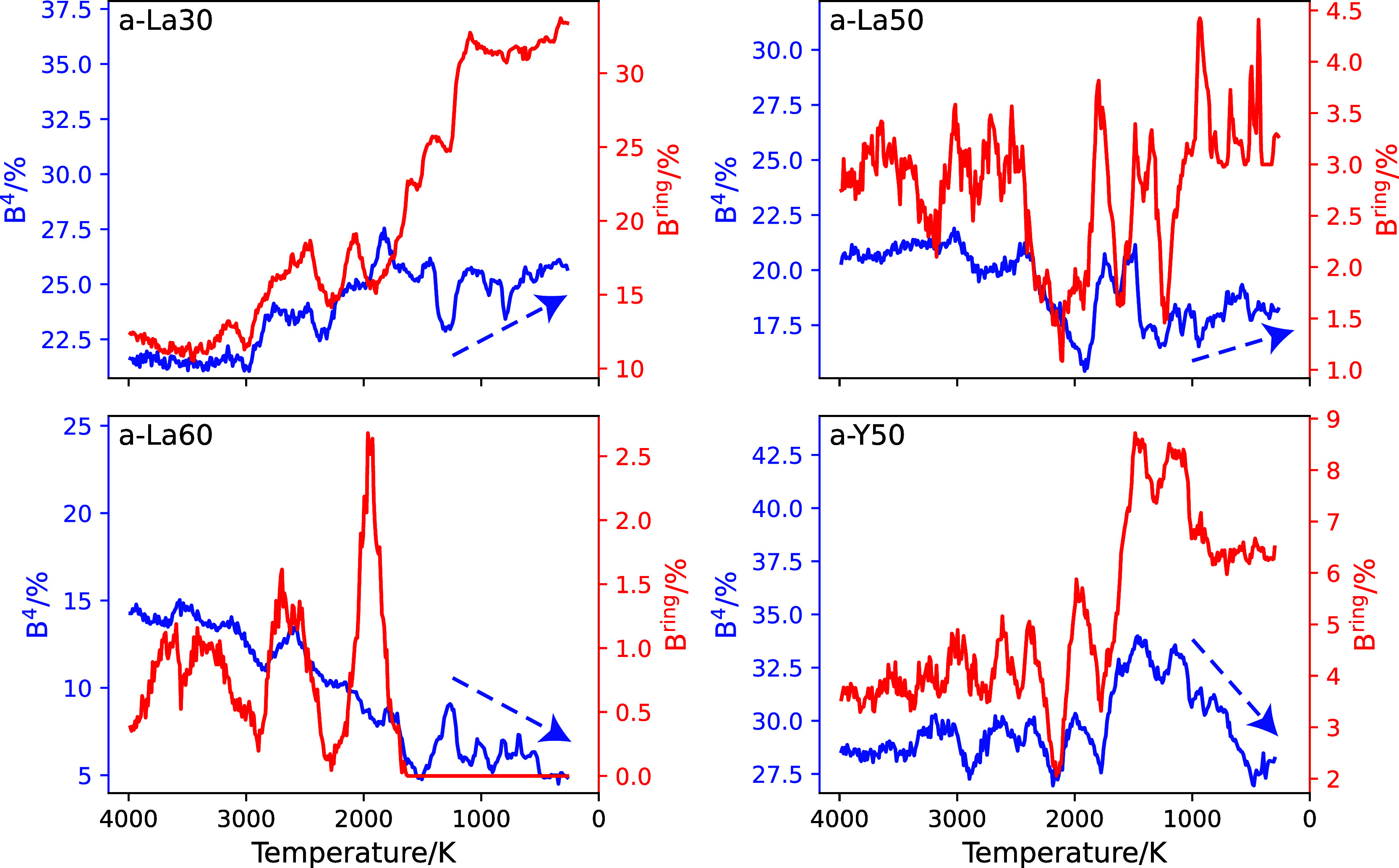
Percentages of B belonging to B^4^ (blue, left-hand axes) and B^ring^ (red, right-hand axes) as a function of temperature. Each panel shows the results for one composition, as indicated in the text in that panel.

The structural changes during the cooling process were evaluated using the boron-unit assignments in Figure 5 at each time step. The temperature-dependent unit population was averaged over a 10-K window to improve statistical accuracy. The percentages of B^4^ atoms are plotted with blue lines in Figure 7 (left-hand axes) as a function of temperature. The formation and cleavage of B–O bonds occur repeatedly over short periods of time, allowing O and B to diffuse freely in the molten state. It should be noted that the percentage of B^4^ in the molten state fluctuates significantly due to this dynamic behavior and the limited number of boron atoms in the cell. Despite these variations caused by the small number of boron atoms, a temperature-dependent trend in the B^4^ percentage can still be discussed. The trends of their percentage changes from 1500 to 300 K are indicated by the dotted lines in Figure 7. For the a-La30 and a-La50 systems, the cooling process leads to a weak increase in B^4^; conversely, the percentage of B^4^ in a-La60 glass shows an unchanged or slightly decreasing trend.

The structural changes of binary sodium borates over the range from room temperature to 1473 K were evaluated by in situ Raman scattering experiments.^40^ For a 15 Na_2_O-85 B_2_O_3_ glass, the B^4^ percentage in the melt was 5% less than that in the solid; a more pronounced B^4^ decrease of about 30% in the glass melt was found in alkali-rich 25 Na_2_O-75 B_2_O_3_ glass. Intense thermal excitation for atomic motion in the molten state favors the B^3^ structure, with O^1^ as a charge compensator of the alkali cation rather than forming B^4^. Despite the low amount of B_2_O_3_ in a-La30 compared to 25 Na_2_O-75 B_2_O_3_ glass, a structural change according to this mechanism was not found. For trivalent REs with a large ionic radius (1.03–1.27 Å for 6–10 coordination) compared to Na^+^ (0.99–1.18 Å for 4–8 coordination),^63^ this mechanism does not make a dominant contribution, and the change in the B^4^ percentages for the melt and solid glass are as small as ∼3%. Interestingly, the change in the B^4^ percentage during the cooling process for a-Y50 glass was as high as ∼7%; this suggests that Y^3+^ has a similar role to that of Na^+^ in the melt. In other words, this behavior is associated with the higher ionicity of Y^3+^ compared to La^3+^, similar to Na^+^.

The formation of 3-rings was monitored by the boron percentages participating in 3-rings (B^ring^), as shown by the red lines in Figure 7 (right-hand axes). An increase in B^ring^ during the cooling process from 1500 to 300 K was found in a-La30 glass. Because the increase in B^ring^ is greater than the change in B^4^, 3-ring formation is attributed to the interconnected reaction of three B^3^s. At higher temperatures, it is suggested that the formation of 3-rings is suppressed by the active atomic motion. For the glasses of other compositions in this study, it is difficult to discuss the changes in B^ring^ during the cooling process due to the large fluctuations resulting from the small amount of boron in 3-rings.

**Figure 8:**
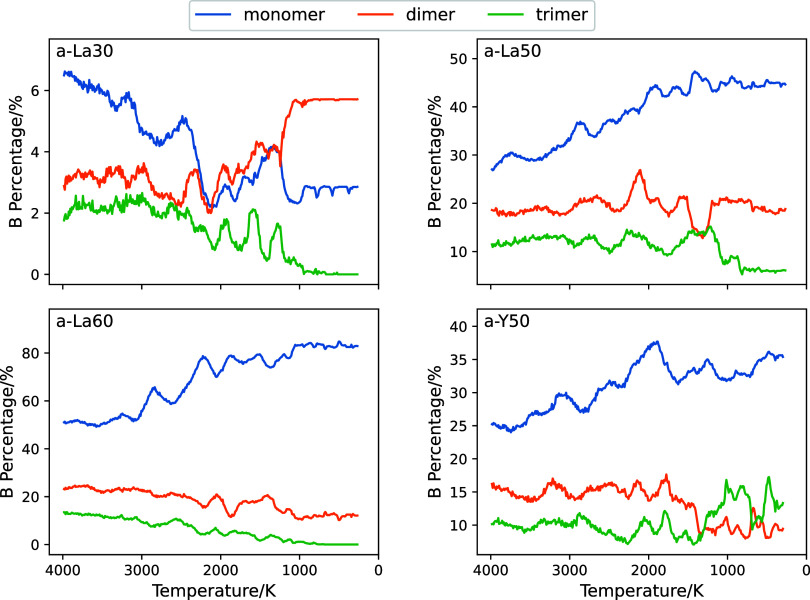
Temperature-dependent percentages of B belonging to monomer, dimer, and trimer units as shown in Figure 5.

The percentages of B assigned to the structural units as indicated in Figure 5 were evaluated as a function of temperature; Figure 8 shows the percentages of B belonging to isolated monomer, dimer, and trimer units. Note that a small amount of B with isolated units—less than 10%—was found in the a-La30glass, because most of the B belongs to polymers. At higher temperatures, thermal excitation may allow more dispersed structural units, and it is possible that unstable structures comprising the units surrounded by blue dashed lines in Figure 5 may be formed.

Although a slight increase in the percentage of B monomers was observed during the cooling process from 1500 to 300 K, the degree of this increase was only about 5%. This result is associated with the fact that linkage reactions between isolated units—such as the formation of a dimer from two monomers—are rare events. The reaction events during the quenching process were clarified in detail by analysis using transition matrices.

The reaction events during the quenching process were counted based on the structural assignment of boron units. Reactive analyses based on MD trajectory data have previously been applied to explore the chemical reactivity and pathways for organic compounds^64,65^; here, a reaction event for RE borate glass melts was defined as a change in the assigned boron units before and after one AIMD step (1 fs). Boron units missing O corresponding to the transition state, such as B1O2 in Figure 5, were additionally found in the melt.

A reaction-event analysis was performed for all data within the temperature range from 1500 to 300 K; the numbers of units recognized within this temperature range are summarized in Table S2. The structural units consisting of isolated 3-rings (B3O6-ring, B3O7-ring, and B3O9-ring) were observed rarely in the glass melts. Note that these structures are not present in the solid glasses. Isolated boron units missing oxygen in monomers, dimers, and trimers (B1O2, B2O4, and B3O5) were also observed only in the melts and are predicted to be the transition units when structural recombination occurs. Dimers with two linked B^4^ units (B2O7) were found primarily in the a-La60 glass melt. This structure has a formal charge of −8*e*, as calculated from the number of O^1^ and the valence electrons of boron. Such an excess negative charge in a dimer unit will lead to instability, except in glass systems with abundant REs. Although double-quantum ^11^B NMR has revealed the BO_4_–BO_4_ linkage in alkali borosilicate glass,^66,67^ the B2O7 dimer with two BO_4_ units is unlikely to be present in RE-rich borate glass. Nonetheless, a trimer consisting only of B^4^ (B3O10) was present in the a-La60 glass; this unit can become more stable by forming a trimer.

**Figure 9:**
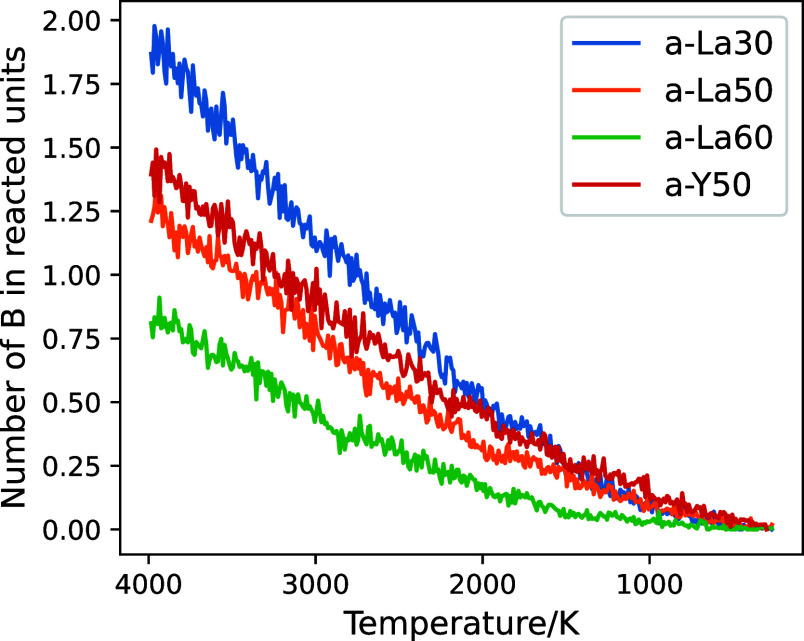
Numbers of boron atoms belonging to reacted units per AIMD step (1 fs) as a function of temperature.

Figure 9 shows the numbers of boron atoms in the reacted units as a function of temperature, in which “reacted units” means those whose label is changed between AIMD steps. The number of boron atoms belonging to reacted units was reduced in the lower-temperature range due to a decrease in the activity of chemical reactions. The number of boron atoms undergoing reaction events is 0.25–0.50 in the glass melts at 2000 K. It can therefore be said that the number of reaction events per step is sufficiently small, meaning that the temporal resolution is sufficient to capture all chemical reactions occurring during the cooling process.

The numbers of boron atoms undergoing unit transitions resulting from reaction events between AIMD steps can be represented as a reaction matrix. The reaction matrices for the a-La30, a-La50, a-La60, and a-Y50 glasses are shown in Figures S3–S6. The reaction events from A to B are represented as rows and columns corresponding to the structural units, where A and B each represent any structural unit in Figure 5. The number of A → B transitions, *N*_A→B_, is normalized by the total number of transitions from A to any unit, ∑ *N*_A→∀_:
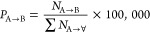
5where 100,000 is a coefficient to avoid excessively small numbers, and the values of *P*_A→B_ are displayed in the cells of the reaction matrix to indicate the probability of a transition event from A to B. In Figures S3–S6, rows and columns corresponding to units observed only in the molten state are filled with beige. The diagonal cells of the reaction matrix are equivalent to the number of steps that remained stable for one AIMD step; conversely, the off-diagonal cells indicate the numbers of boron atoms in units transitioned from A to B via reaction events. The red bars in the cells in Figures S3–S6 are drawn to visualize the relative frequencies of the reaction events; their visualization was performed for each row to rank the probabilities of the reaction destinations.

A low reaction probability from the polymer to isolated B1O3, B2O5, and B3O7 was observed in the a-La30 glass. Frequent reactions of polymers between those with and without a 3-ring were observed in all systems. In a-La50, this reaction can also be seen in the video attached in the Supporting Information (chain-ring.mp4), in which polymers with and without 3-rings are depicted with yellow and red bars, respectively, at each time step. The formation and destruction of units with 3-rings is achieved by the mutual reaction between B^3^ and B^4^.

**Figure 10:**
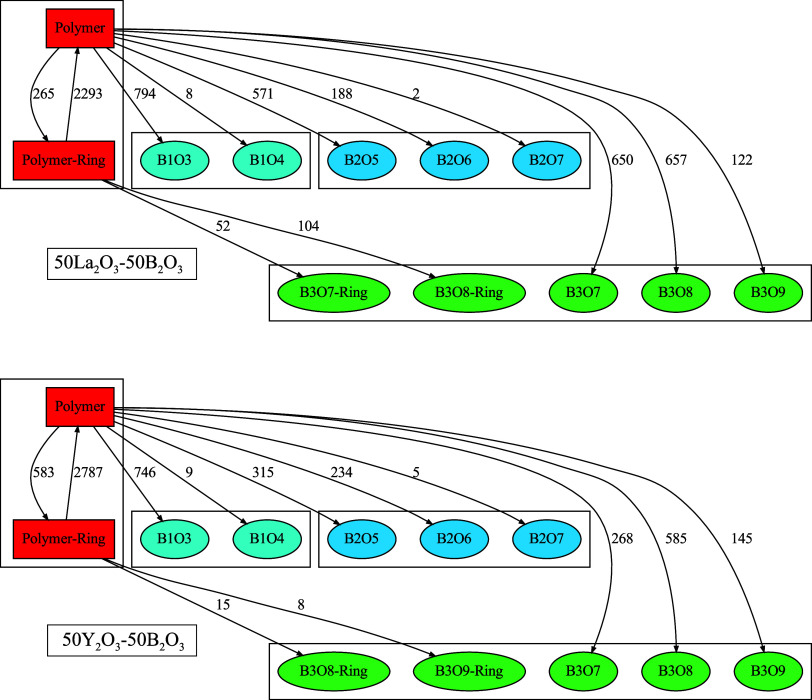
Network graphs of the reactions detected in a-La50 (upper) and a-Y50 (lower). Selected reaction paths for the exchange between polymers and polymer-ring structures and one-way reactions to isolated units are visualized. The nodes for monomers, dimers, trimers, and polymers are filled with different colors. The arrows between nodes indicate reaction paths along with their probabilities.

Interestingly, the reaction to isolated units from polymers with 3-rings was found to be unlikely. In other words, the formation of isolated units (monomers, dimers, and trimers) tends to arise from polymers without 3-rings. This aspect was illustrated by means of the NetworkX with Python package^68^ based on the reaction matrix in Figures S3–S6. Figure 10 shows network graphs for the a-La50 and a-Y50 glasses with the selected reaction paths related to the polymer. The nodes for monomers, dimers, trimers, and polymers with different colors and frames are connected with arrows indicating the reaction probability corresponding to each reaction path as defined in eq 5.

The network graphs clarify the reaction paths of polymers to isolated units or polymer-ring structures. Interestingly, the reaction paths from polymer-ring structures to isolated monomers or dimers were rare events compared with the change from polymers to isolated units. This finding is associated with the stabilization of a polymer involving 3-ring formation. Although all the reaction paths in the a-La50 and a-Y50 glasses exhibit very similar probabilities, the probability of reaction from polymer to B3O7 trimer in a-Y50 is less than that in a-La50. The probability of a mutual reaction between a polymers and polymer-ring structures in a-Y50 is higher than that in a-La50. This difference can be attributed to the presence of higher 3-ring contents in the a-Y50 glass compared to a-La50.

### RE Coordination Structure

**Figure 11:**
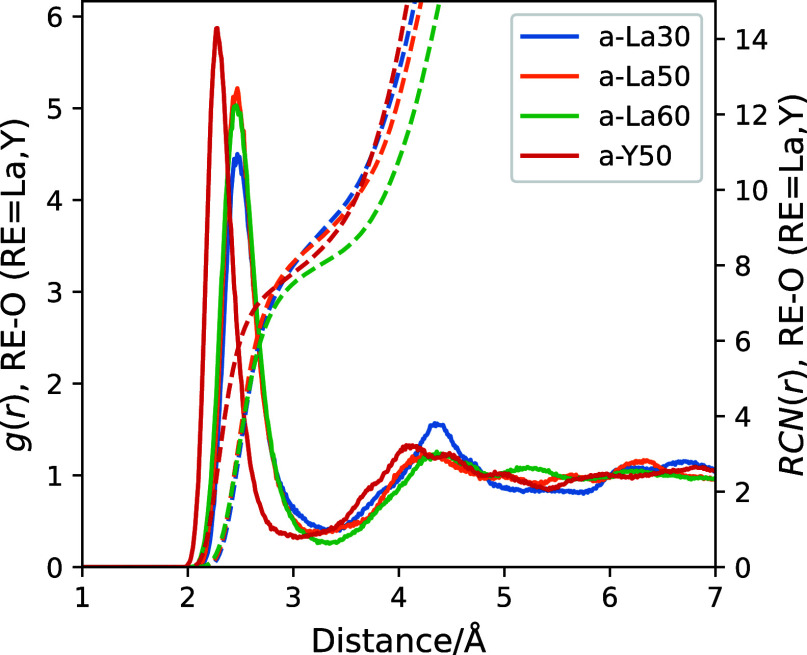
Radial distribution functions [*g*(*r*), solid lines] and running coordination numbers [RCN(*r*), dashed lines] of RE–O (RE = La, Y) estimated from AIMD-derived RE glass structures at 300 K.

Figure 11 shows plots of *g*(*r*) and RCN(*r*) of RE–O (RE = La, Y) for the four glass systems at 300 K. The RE–O *g*(*r*) plots in Figure 11 indicate first-peak maxima at 2.633, 2.600, 2.586, and 2.394 Å for a-La30, a-La50, a-La60, and a-Y50, respectively. The cutoff bond distances for La–O and Y–O bonds were set to 3.30 and 3.05 Å, respectively, as determined from the first-minimum position in Figure 11. The running coordination numbers of RE from RCN(*r*) at these points are 8.974, 8.817, 8.076, and 7.781 for a-La30, a-La50, a-La60, and a-Y50, respectively.

**Figure 12:**
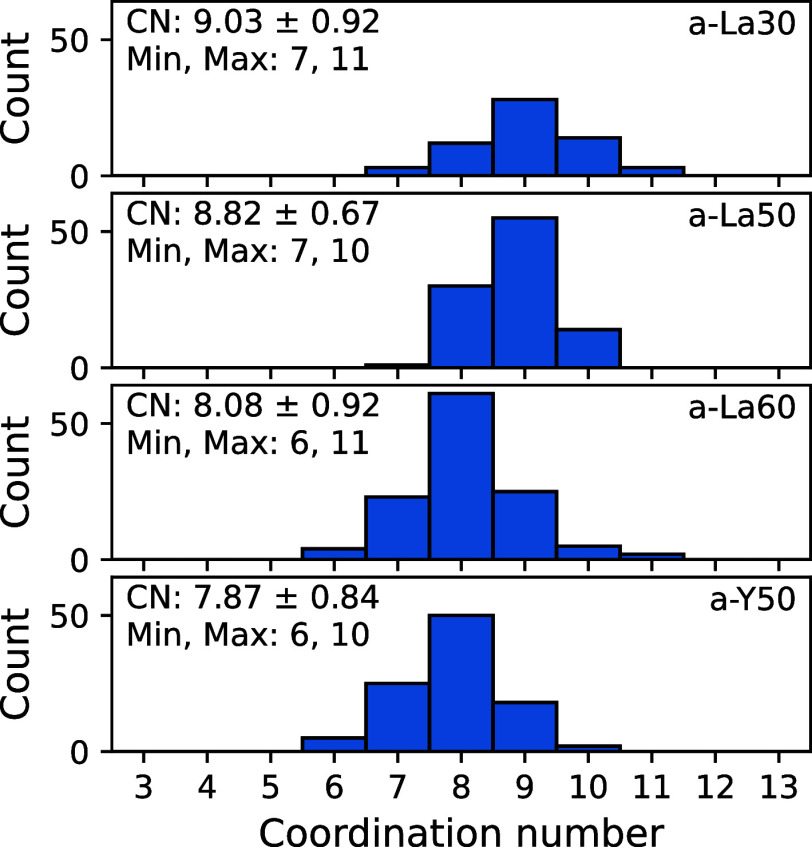
Histograms of coordination numbers of REs in four glass systems. The text in each panel shows the mean and standard deviation; maximum and minimum coordination numbers are also shown.

The coordination structures of the REs in the glasses were extracted from the optimized glass structures for analysis considering the periodic boundary conditions of the simulation cell. All coordination structures are individually displayed in Figures S7–S10. Figure 12 shows histograms of the coordination numbers calculated from the extracted coordination structures along with statistics. The histograms of coordination numbers each have a symmetrical distribution, and for the La_2_O_3_–B_2_O_3_ glass system, the peak position changes to smaller values with increasing La_2_O_3_. The RE coordination number for a-Y50 is lower than that for a-La50. It is expected that the extended coordination sphere of La^3+^ leads to a higher coordination number than that for Y^3+^.

A comprehensive analysis of the symmetry and shape of each extracted RE coordination structure was undertaken by means of the inertia tensor, spherical harmonics, and Voronoi tessellation analysis. These analyses are now briefly described here.

The inertia tensor was calculated from the La and O positions (*x*_*i*_, *y*_*i*_, *z*_*i*_ in Å) from the centroid (*G*) and mass (*m*_*i*_ in g/mol) for the coordination structure. This analysis successfully represents the shape of the ring structure in the glass.^69^ The inertia matrix is defined as
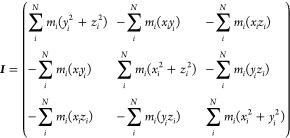
6where ∑ _*i*_^*N*^ is the summation for La and all *N* coordinated oxygen atoms. The principal-axis conversions into the diagonal matrix elements, *I*_*a*_ ≤ *I*_*b*_ ≤ *I*_*c*_, were obtained by solving the eigenvalue problem to represent the degree of sphericity. The relative values of *I*_*a*_, *I*_*b*_, and *I*_*c*_ can be used as an index for the coordination shape:
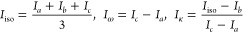
7These parameters derived from the inertia tensor can be classified into size (*I*_iso_), asymmetry (*I*_ω_), and oblate or prolate ellipsoids (*I*_κ_).

The extent of symmetry can also be represented by a series of coefficients of spherical harmonic functions, *Y*_*lm*_(θ, ϕ):
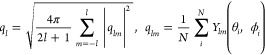
8where *lm* are combinations of *l* and *m* for spherical harmonic functions with integer *l* and *m* for the degree and order of the harmonic; *Y*_*lm*_(θ, ϕ) can be calculated from the coordinated O positions in polar coordinates (θ and ϕ) for the coordination system with the RE at the origin. The parameter *q*_*l*_ can be made rotationally invariant by summing *m* for each RE coordination structure. The coefficients of the spherical harmonics were considered up to *l* = 6. This parameter correlates with the electric-field gradient of the central ion, and it is known as the quadrupolar coupling constant obtained by solid-state NMR experiments.^48^

Voronoi tessellation analysis was applied to each RE coordination structure to establish the local atomic arrangements in the amorphous materials.^70^ The Voronoi cells were made from Voronoi nodes (oxygen positions) for each RE coordination structure; the Voronoi cell volume (*V*_vol_) and indices (*V*_*p*_) represent the topology information on the Voronoi polyhedron. The value of *V*_p_ is the number of edges p on the faces in the Voronoi cell. The number of edges on a face was counted when the vector from the RE to oxygen crossed the face.

Although it is difficult to interpret a set of these parameters intuitively, the model polyhedron structure for a defined coordination number can be used to evaluate the degree of the shape and its symmetry. The positions of *n* point charges on the spherical surface were optimized to minimize the Coulombic energy. This optimization problem is known as the Thomson problem.^71,72^ Model structures with *n* = 6–12 were derived from numerical minimization of the Coulombic energy by optimizing charge positions to compare with all the AIMD-derived structures in Figures S7–S10. These structures have the same minimum Coulombic energy reported in a previous study.^72^ The model polyhedron structures are displayed in Figure S11. All the parameters for the inertia tensor, spherical harmonics, and Voronoi tessellation analysis for the model structure are summarized in Table S3 for comparison purposes.

Table 3 lists the mean values of all of the representative parameters obtained from the extracted coordination structures; the mean coordination number (CN), bond distance (*R̅*), and RE distance from *G* are also included. As reference parameters, the results from crystalline LaBO_3_ and YBO_3_ are also listed in Table 3. Two Y sites (8e and 4b in Wyckoff notation) in YBO_3_ were individually estimated.

**Table 3:** Set of Parameters to Represent the RE Coordination Structures for Four Glass Systems and Two Crystalline Phases^a^

	CN	*G*	*R̅*	*I*_iso_	*I*_ω_	*I*_κ_
a-La30	9.03	0.149	2.627	11889.8	2809.0	–0.018
a-La50	8.82	0.140	2.595	11132.1	2489.6	–0.040
a-La60	8.08	0.133	2.576	9705.2	2301.5	–0.028
a-Y50	7.87	0.154	2.395	6531.5	1531.5	–0.021
LaBO_3_	8	0.158	2.566	9305.3	2461.6	0.059
YBO_3_ (8e)	8	0.099	2.334	6275.4	728.2	0.140
YBO_3_ (4b)	8	0.000	2.335	6309.7	1472.6	–0.031

	*q*_1_	*q*_2_	*q*_3_	*q*_4_	*q*_5_	*q*_6_
a-La30	0.094	0.105	0.158	0.284	0.424	0.369
a-La50	0.090	0.098	0.151	0.283	0.456	0.374
a-La60	0.093	0.108	0.174	0.342	0.472	0.362
a-Y50	0.092	0.108	0.177	0.349	0.495	0.353
LaBO_3_	0.117	0.120	0.139	0.432	0.346	0.444
YBO_3_ (8e)	0.071	0.070	0.155	0.465	0.203	0.551
YBO_3_ (4b)	0.000	0.135	0.000	0.500	0.000	0.590

	*V*_vol_	*V*_3_	*V*_4_	*V*_5_	*V*_6_	*V*_7_
a-La30	33.313	0.183	3.300	4.867	0.667	0.017
a-La50	31.747	0.070	3.350	5.090	0.310	0.000
a-La60	28.504	0.150	3.808	3.933	0.192	0.000
a-Y50	22.386	0.160	3.880	3.760	0.070	0.000
LaBO_3_	26.691	1	6	1	0	0
YBO_3_ (8e)	20.583	0	4	4	0	0
YBO_3_ (4b)	20.608	0	6	0	2	0

a The parameters in the glass systems are the average values of all the coordination structures in the overall structure. Here: CN, *G* in Å, and *R̅* in Å are the coordination number, distance of the RE from the centroid, and mean RE–O distance; *I*_iso_ in g/mol·Å^2^, *I*_ω_ in g/mol·Å^2^, and *I*_κ_ are derived from the inertia tensor; the values of *q*_*l*_ (*l* = 1–6) are calculated from spherical harmonics; *V*_vol_ is the cell volume in Å^3^; and *V*_3_–*V*_7_ are the indices of the Voronoi cells.

The mean RE coordination number of the La_2_O–B_2_O_3_ glasses decreases with increasing La_2_O_3_, as shown in Figure 12. Increasing La_2_O_3_ results in smaller *R̅* values, and it also decreases *I*_iso_ and *V*_vol_, which are related to the volume occupied by the RE in the coordination structure. For La_2_O_3_–B_2_O_3_ glass, the degree of asymmetry interpreted from *I*_ω_ decreases with increasing La_2_O_3_ content, meaning that the coordination structure becomes more spherical. The reference structures from the solution of the Thomson problem in Table S3 show much smaller *I*_ω_ values for CN = 6–12 (0–784.1); hence, the RE coordination structure in the glass is far from stable when considering Coulombic energy alone. The oxygen not bonded to boron, as shown by O^0^ species in Figure 4, is coordinated only to La in a stable position. As a result, a highly spherically symmetric coordination structure like the model structure is expected to form in glasses with high La_2_O_3_ content.

The *q*_*l*_ parameters can also capture the degree of spherical asymmetry, which differ from the reference models, as shown in Table S3. The *q*_4_ and *q*_5_ parameters in three La_2_O_3_–B_2_O_3_ glass systems have similar values that are comparable to the values for the reference models with CN = 8. The geometry of the structure with CN = 8 is a square antiprism polyhedron (a prismatic uniform polyhedron) with a *D*_4*d*_ point group. The significant difference between a-La50 and a-Y50 was found in the *I*_ω_ value. The RE coordination structure of a-Y50 has the lowest degree of asymmetry among the four glasses as predicted from *I*_ω_. The decreasing *V*_5_ and increasing *V*_4_ values in a-Y50 compared to a-La50 also suggest a CN change related to the reference models from CN = 9–12. Here, the trend due to the increase in CN corresponds to the parameter change between a-La50 and a-Y50.

The symmetry parameters (*I*_ω_, *q*_*l*_, and *V*_*n*_) for the coordination structure in crystalline LaBO_3_ were almost equivalent to those for a-La50 glass; however, the volume occupied by the RE expected from *I*_iso_ and *V*_vol_ was reduced in comparison to a-La50 glass. This is associated with the difference in the effect of packing on polyhedral coordination structures in ordered and disordered systems.

A significantly small *I*_ω_ value and characteristic Voronoi index are found in the 8e and 4b sites with CN = 8 for Y in crystalline YBO_3_. From visual inspection, the Y coordination structure of the 8e and 4b sites in the YBO_3_ crystal seems to be a square prismatic geometry rather than the square antiprism polyhedron corresponding to the reference model. Conversely, the polyhedral structure in crystalline LaBO_3_ is understood to have a lower degree of spherical symmetry, as expected from the higher *I*_ω_ value, even in ordered crystals. The degree of symmetry of the La coordination structure in the crystal is not much different from that of a-La50 glass. The novel ring structure consisting of three B^4^ formed in the YBO_3_ crystal, as shown in Figure S1, can be achieved with a specially ordered structure including Y with square prismatic geometry.

The increase in spherical symmetry expected from the lower *I*_ω_ with higher La_2_O_3_ content is associated with a decrease in the B^4^ percentage for the three La_2_O_3_–B_2_O_3_ glass systems. La^3+^, as a multivalent cation, interacts electrostatically with negatively charged O^1^ or B^4^. When the charge is compensated at the central B^4^ atom in the tetrahedron, the oxygen atoms at the vertices are less likely to adopt a configuration that promotes high spherical symmetry. Consequently, the reduction in B^4^ content correlates with the increased spherical symmetry of the La coordination environment.

The electronic properties of the coordination structure were analyzed by means of Bader partial charge using a code developed by the Henkelman group.^73,74^Table 4 lists the mean Bader partial charges of each element in the four glass and crystalline systems; these vary by only about 0.1*e* in both the glass and the crystal. Interestingly, the boron partial charge in YBO_3_, which consists of only B^4^, is not much different from that of B^3^ in LaBO_3_. The boron formal partial charge predicted from the coordination number does not correspond to the calculated Bader partial charge. The La^3+^ and Y^3+^ in the crystal have greater Bader charges than those in the glass, when comparing the sample composition.

**Table 4:** Mean Bader Partial Charges of Each Element in the Glass Systems and Two Crystalline Phases^a^

	La	Y	B	O
a-La30	2.196		2.289	–1.508
a-La50	2.138		2.267	–1.468
a-La60	2.112		2.256	–1.446
a-Y50		2.203	2.267	–1.490
LaBO_3_	2.186		2.242	–1.476
YBO_3_ (8e)		2.217		
YBO_3_ (4b)		2.229		
YBO_3_			2.289	–1.503

a The partial charge of non-equivalent Y at the 8e and 4b sites in crystalline Y_2_O_3_ are listed in separate rows.

**Figure 13:**
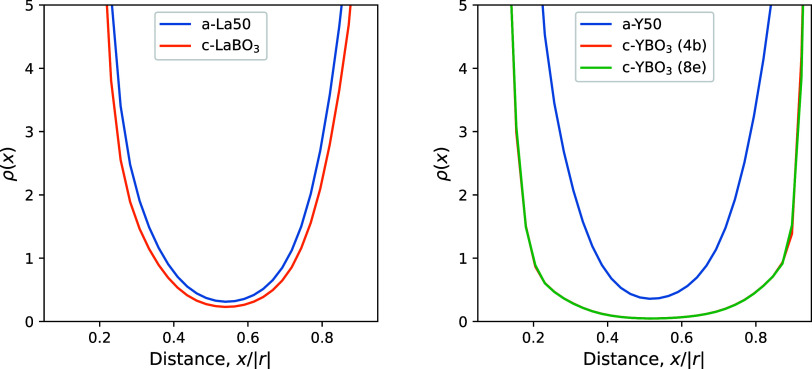
Mean electron distributions along RE–O bonds (RE = La, Y) for (left) La_2_O_3_–B_2_O_3_ and (right) Y_2_O_3_–B_2_O_3_ systems. The crystal data are taken from our previous study. Note that the orange line corresponding to the 4b site for YBO_3_ is invisible because it almost overlaps with the 8e sites over the entire *x* range.

The space distributions of electrons for all RE–O bonds were analyzed using line profiles of electron density ρ(*x*), as calculated from Gaussian-cube format data obtained from wave functions. The value of ρ(*x*) is normalized by the total number of electrons in the cell and the voxel volume. The line segment from RE to O was divided into 40 points corresponding to *x*, and the electron density at each *x* value was obtained by interpolation from the surrounding voxels. The mean electron distributions along the RE–O bonds for the a-La50 and a-Y50 glass systems are shown in Figure 13 along with the crystalline data reported in our previous study.^29^ The results of ρ(*x*) for a-La30 and a-La60 are shown in Figure S12. Note that to compare the distributions, *x* is the relative position normalized by each RE–O bond length |*r*|.

The minimum position obtained in the a-Y50 glass system was shifted toward a shorter position compared to the three La_2_O_3_–B_2_O_3_ glass systems. This is due to the relatively expanded coordination sphere of La^3+^ compared to that of Y^3+^, as indicated by the bond distances and occupied RE volumes listed in Table 3. Almost identical electron distributions along La–O bonds were obtained for the a-La50 glass and LaBO_3_ crystal systems. No special changes in La_2_O_3_ content were observed, as shown in Figure S12. Nonetheless, a clear difference was found in the distribution between a-Y50 glass and YBO_3_ crystal.

The electron distribution in the YBO_3_ crystal is clearly biased toward the ionic side (*x* = 0 or 1) compared to the distribution in a-Y50 glass; it is expected that the electron distribution in the YBO_3_ crystal is polarized toward the Y or O ions compared to the distribution in a-Y50 glass. This behavior corresponds to a higher degree of ionic rather than covalent bonding, and this special form found in YBO_3_ crystals completely disappears in the a-Y50 glass. Such a structural change suggests that a ring structure composed only of B^4^ cannot survive in the random arrangement of a-Y50 glass. As a result, the unique 3-ring structure in YBO_3_ crystal or an analogous pair of B^4^ dimers cannot be observed in the glass.

## Conclusions

RE-rich borate glasses were modeled by means of AIMD simulations following a melt-quenching route. Isolated borate units and chain structures involving 3-rings were found in the AIMD-derived structures. The structural factors and solid-state ^11^B NMR data were reasonably consistent with the AIMD-derived structures. A variety of boron units identified by B–O bonds were classified into monomers, dimers, trimers, and polymers depending on their number of boron atoms. The main isolated boron units in a-La50 were monomers and dimers with B^3^, and their abundance increased during the melt-quenching process. A dimer consisting of two B^4^ present in the melts was not found in the glasses.

The formation mechanisms of these boron units were analyzed using transition matrices, which were defined as the unit changes either side of an AIMD step. Transitions between polymers with and without 3-rings were frequently observed in the melt; the reaction routes from polymer units with 3-rings to isolated units are less likely in the melt; this is associated with the stabilization of polymers by 3-ring formation.

Comprehensive analyses to characterize RE coordination structures were performed with a view to understanding the differences between RE glasses and crystals. The coordination structures were represented by the inertia tensor, spherical harmonics, and Voronoi cells. The parameters derived from these analyses were compared with the model structure obtained from solving the Thomson problem with CN = 6–12. The degree of disorder and asymmetry was detected from *I*_ω_, which was found to take greater values than the model structure. The RE coordination structure in the glass is disordered compared to the model structure with CN = 8, which is assigned to the square antiprism polyhedron. Clear differences were found in the electron distributions for Y–O bonds between the YBO_3_ crystal and a-Y50. The characteristic polarization seen in the YBO_3_ crystal disappears in the a-Y50 glass, and the novel 3-ring structure in YBO_3_ is no longer formed in the a-Y50 glass. It was found that a series of structural and formation properties in terms of the “degree of disorder and symmetry” are linked to each other.

Analyses focusing on local structure and formation mechanisms will be useful for describing the nature and properties of glasses. Understandings of the origin of glasses in the group with CN > 6 polyhedra, such as PbO glass, will be advanced by the approaches used in this study.

## Data Availability

All AIMD data and analytical scripts can be provided upon request.

## Supporting Information Available

Reactions of chains with and without 3-rings (chain-ring) (MP4)
Crystalline LaBO_3_ and YBO_3_ structures; experimental and simulated ^11^B magic-angle spinning (MAS) NMR data for a-La30; numbers of boron atoms assigned to boron units in Figure 5 during AIMD runs in the temperature range from 1500 to 300 K; reaction matrices for a-La30, a-La50, a-La60, and a-Y50; visualizations of all RE coordination structures extracted from AIMD-derived glass structures; visualizations of the reference model coordination structures obtained from the solution of the Thomson problem; shape and symmetry parameters corresponding to Table 3 for the model structures; and mean electron distributions along RE–O bonds for the a-La30, a-La50, a-La60, and a-Y50 glasses (PDF)
