# Supplementary material for: Ab Initio Molecular Dynamics Study of Trivalent Rare Earth Rich Borate Glasses: Structural Insights and Formation Mechanisms
Source: J Phys Chem B. 2024 Nov 11;128(47):11800–13. doi: 10.1021/acs.jpcb.4c05039 (PMC11613562; doi:10.1021/acs.jpcb.4c05039)
Supplement: Supplementary file 2 — jp4c05039_si_002.pdf [file jp4c05039_si_002.pdf]

Supporting Information: Ab Initio Molecular Dynamics Study of Trivalent Rare Earth Rich Borate Glasses: Structural Insights and Formation Mechanisms

Takahiro Ohkubo,[*,†] Shunta Sasaki,[‡] Atsunobu Masuno,[‡] and Eiji Tsuchida[¶]

†*Graduate School of Engineering, Chiba University, 1-33 Yayoi-cho Inage-ku, Chiba 263-8522, Japan*

‡*Department of Material Chemistry, Graduate School of Engineering, Kyoto University, Kyotodaigaku-Katsura, Nishikyo-ku, Kyoto 615-8520, Japan*

¶*National Institute of Advanced Industrial Science and Technology (AIST), Tsukuba Central 2, Umezono 1-1-1, Ibaraki 305-8568, Japan*

E-mail: ohkubo.takahiro@faculty.chiba-u.jp

Phone: +81 (0)43 2903431. Fax: +81 (0)43 2903431

Crystalline structure

For comparison with the glass structures, the crystalline structures of $LaBO_3$ and YBO_3[S1,S2] were drawn using VESTA,[S3] as shown in Fig. S1. Here, a novel 3-ring structure with three B^4 units in YBO_3 is indicated by black dashed lines.

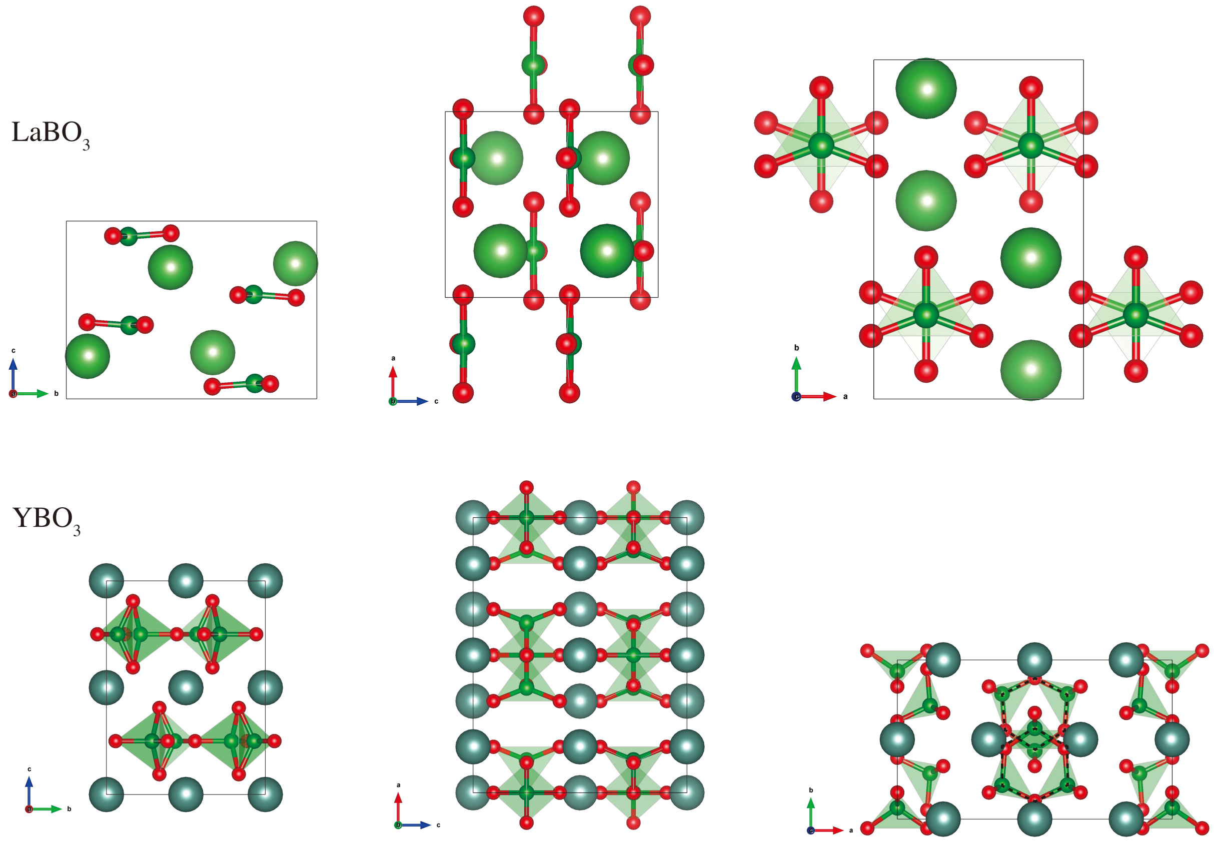

Fig. S1: Crystalline structures of $LaBO_3$ (upper panels) and YBO_3 (lower panels), showing the *bc*, *ac*, and *ab* planes. The 3-ring structure consisting of three B^4 units in YBO_3 is indicated by black dashed lines. The ball and stick colors are B: green; O: red; La: light green; and Y: gray.

^{11}B solid-state NMR

The solid-state ^{11}B magic-angle spinning (MAS) NMR spectrum of a-La30 glass was measured to estimate the boron species (Fig. S2). The ^{11}B MAS NMR spectra of the other glasses (a-La50, a-La60, and a-Y50) were reported in our previous study.[S4] The spectral simulation was performed with three boron components (B^3-nonring, B^3-ring, and B^4), considering second-order quadrupolar interactions. The simulated parameters are summarized in Table S1.

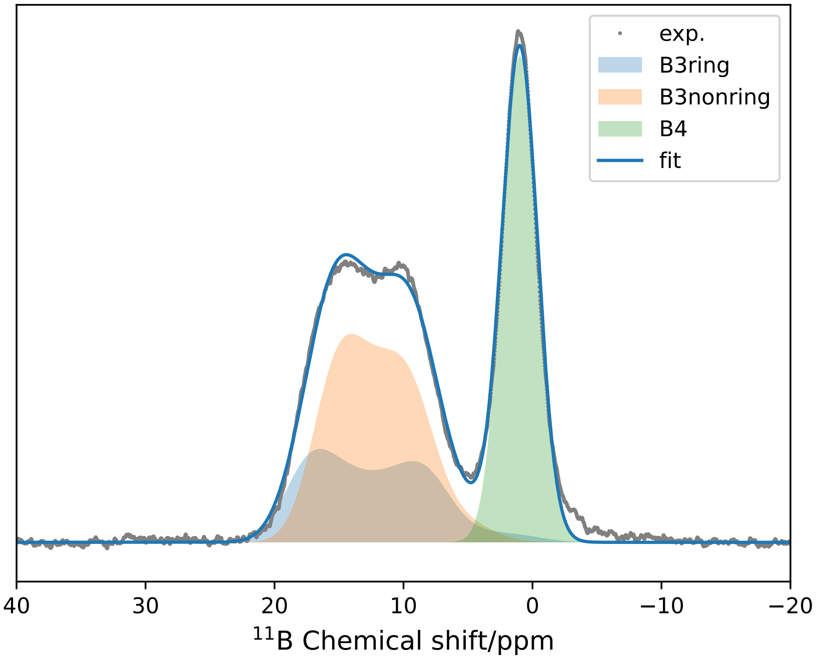

Fig. S2: Experimental solid-state ^{11}B magic-angle spinning NMR spectrum and simulated lines of a-La30.

Table S1: Fitting parameters obtained from spectral simulation of the ^{11}B MAS NMR spectrum of 30 La_2O_3-70 B_2O_3 glass. Here, CS, C_q, η, and BF are the isotropic chemical shift in ppm, the quadrupolar coupling constant, the asymmetry parameter, and the Gaussian broadening factor, respectively.

	Pop. (%)	CS (ppm)	C_q (MHz)	η	BF (Hz)
B3ring	24.1	21.2	3.0	0.0	700.0
B3nonring	41.5	18.1	2.5	0.0	700.0
B4	34.4	1.1	0.3	0.0	523.8

Reaction analysis

The numbers of boron atoms assigned to boron units in Fig. 5 of the main text were counted during the AIMD run from 1500 to 300 K, and the results are listed in Table S2.

Table S2: Numbers of boron atoms in labeled units during the AIMD run in the temperature range from 1500 to 500 K. The boron units corresponding to the labels can be found in Fig. 5 of the main text.

Label	a-La30	a-La50	a-La60	a-Y50
B1O2	1613	600	242	260
B1O3	215 721	2 162 925	2 948 328	1 685 693
B1O4		48 258	123 668	100 916
B2O3			2	
B2O4	72	4		
B2O5	349 860	663 474	473 250	299 838
B2O6	856	272 180	78 736	248 490
B2O7		190	2016	394
B3O5	3			
B3O6-Ring	174			
B3O7	45 669	133 779	2517	214 305
B3O7-Ring		459		252
B3O8	828	318 285	46 329	286 401
B3O8-Ring		9543		324
B3O9		71 784	2841	144 435
B3O9-Ring				39
B3O10			9288	
Polymer	4 439 356	1 137 314	151 823	2 048 832
Polymer-Ring	2 025 648	128 205		399 721

The reaction matrices for a-La30, a-La50, a-La60, and a-Y50 are shown in Figs. S3–S6. The reaction events from A to B are represented as rows and columns, where A and B represent any of the boron structural units shown in Fig. 5 of the main text. The numbers in the matrices indicate transition probabilities as defined in Eq. (3) of the main text. The red bars in the cells are drawn to visualize the relative frequencies of the reaction events. Their visualization was performed for each row to rank the probabilities of the reaction destinations. The rows and columns corresponding to the units observed only in the molten state are filled with beige.

	B1O2	B1O3	B2O4	B2O5	B2O6	B3O5	B3O6 Ring	B3O7	B3O8	Polymer	Polymer Ring
B1O2	984762	635		1905				2540		10159	
B1O3	4	998502		11	21				44	1418	
B2O4			944444							55556	
B2O5	12	12		998519				23	89	1335	10
B2O6		7009			990654					2336	
B3O5										666667	333333
B3O6 Ring							965517				34483
B3O7	102			205				990215		9478	
B3O8		13285		26570					956522	3623	
Polymer	3	74	1	96				92	1	999319	414
Polymer Ring							3	1		961	999035

Fig. S3: Reaction matrix of a-La30.

	B1O2	B1O3	B1O4	B2O4	B2O5	B2O6	B2O7	B3O7	B3O7 Ring	B3O8	B3O8 Ring	B3O9	Polymer	Polymer Ring
B1O2	961474	4606			15075			10050		3350		419	5025	
B1O3	1	998808	58		8	447				189		69	418	1
B1O4		2193	997584				13					47	163	
B2O4		250000			250000					250000			250000	
B2O5	13	26			997491	205		18		1269		5	971	3
B2O6	2	3300	2		559	994249				8		1190	689	
B2O7		5263	5263				978947						10526	
B3O7	37	15			81	4		994037	66	343			5418	
B3O7 Ring								13072	954248					32680
B3O8	6	1076			2150	25		171		994350	134	89	1990	7
B3O8 Ring										5030	992455			2515
B3O9		2117	16		31	4235				445		990859	2297	
Polymer	4	794	8		571	188	2	650		657		122	996742	265
Polymer Ring									52		104		2293	997551

Fig. S4: Reaction matrix of a-La50.

	B1O2	B1O3	B1O4	B2O3	B2O5	B2O6	B2O7	B3O10	B3O7	B3O8	B3O9	Polymer
B1O2	967533	6494			25974							
B1O3		999647	111		6	169	2	1		41	2	19
B1O4		3000	996812				71	112				6
B2O3					1000000							
B2O5	10	23	3		999024	106				455		380
B2O6		5823			785	992436	83	401		18	130	325
B2O7		3968	3968			2976	985119	3968				
B3O10		538	1292			2584	1077	991925			2584	
B3O7		1193							974940			23866
B3O8		2411			4676					991653		1260
B3O9		2300	708		1769	3185		8493			982484	1062
Polymer		366	5		1428	212			409	321	29	997229

Fig. S5: Reaction matrix of a-La60.

	B1O2	B1O3	B1O4	B2O5	B2O6	B2O7	B3O7	B3O7 Ring	B3O8	B3O8 Ring	B3O9	B3O9 Ring	Polymer	Polymer Ring
B1O2	942085	11583		19305					1931				25097	
B1O3	1	998138	69	5	554				171		164		898	
B1O4		1078	998755	6	3						12		142	3
B2O5	14	21		996302	104				1565		9		1984	
B2O6		3718		134	991847	26					2256		2018	
B2O7		2538	2538		10152	959391							25381	
B3O7		9		4			996949	15	194		4		2824	
B3O7 Ring							11905	976191						11905
B3O8		803	3	1596	3		124		993606	26	121		3718	
B3O8 Ring									27778	953704				18519
B3O9		1953	19	38	3907				244		991689		2150	
B3O9 Ring												923077		76923
Polymer	3	746	9	315	234	5	268		585		145		997107	583
Polymer Ring										15		8	2787	997190

Fig. S6: Reaction matrix of a-Y50.

RE symmetry

All of the RE (La and Y) coordination structures extracted from the AIMD-derived glass structures are displayed in Figs. S7–S10. The directions of the longest and second-longest RE–O bonds are aligned parallel to the z axis and the xz plane for ease of visual inspection by rotational operation. Green, gray, and red balls indicate La, Y, and O, respectively.

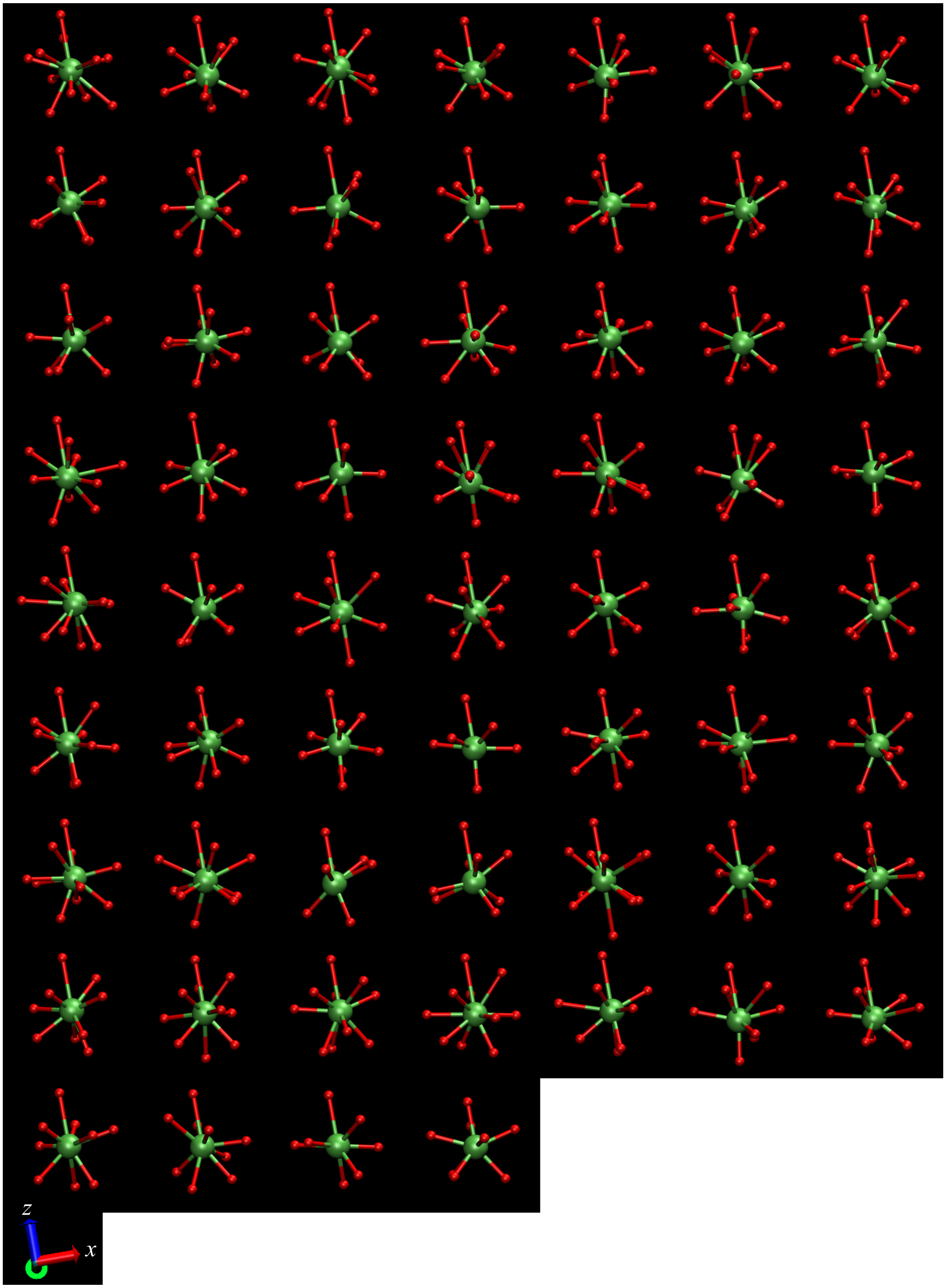

Fig. S7: All extracted La coordination structures for a-La30.

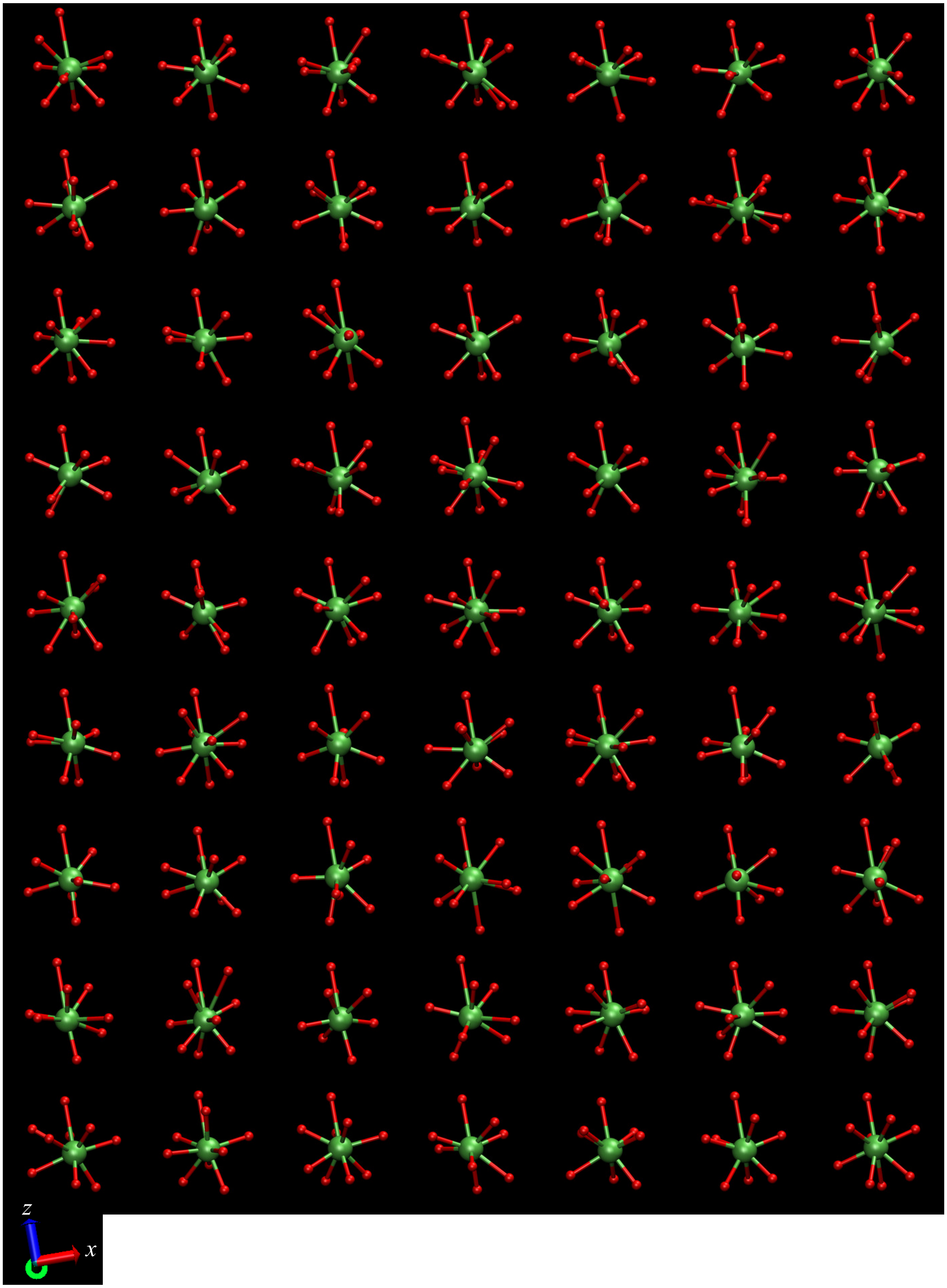

Fig. S8: All extracted La coordination structures for a-La50.
(continued on next page)

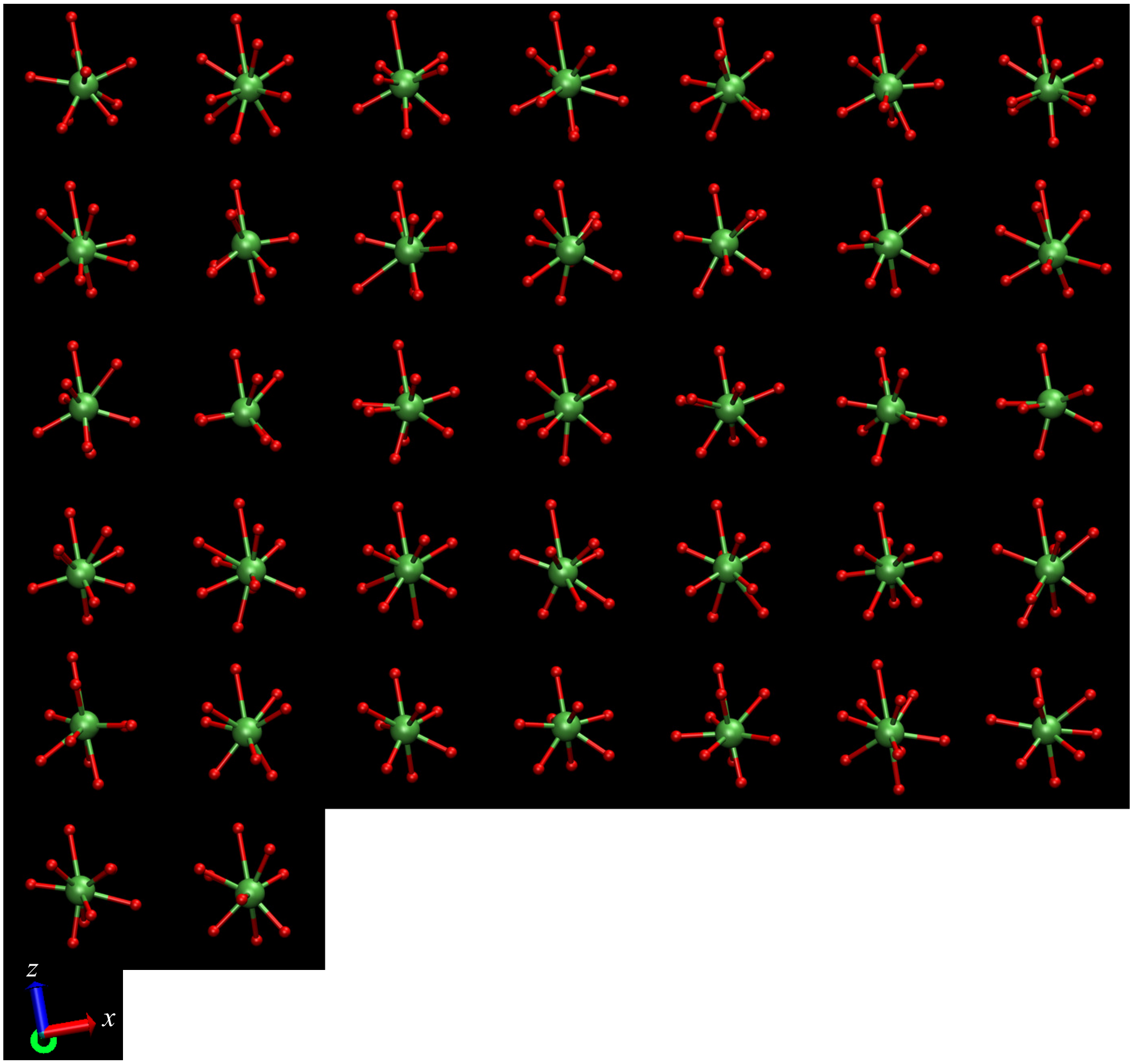

(Fig. S8 end)

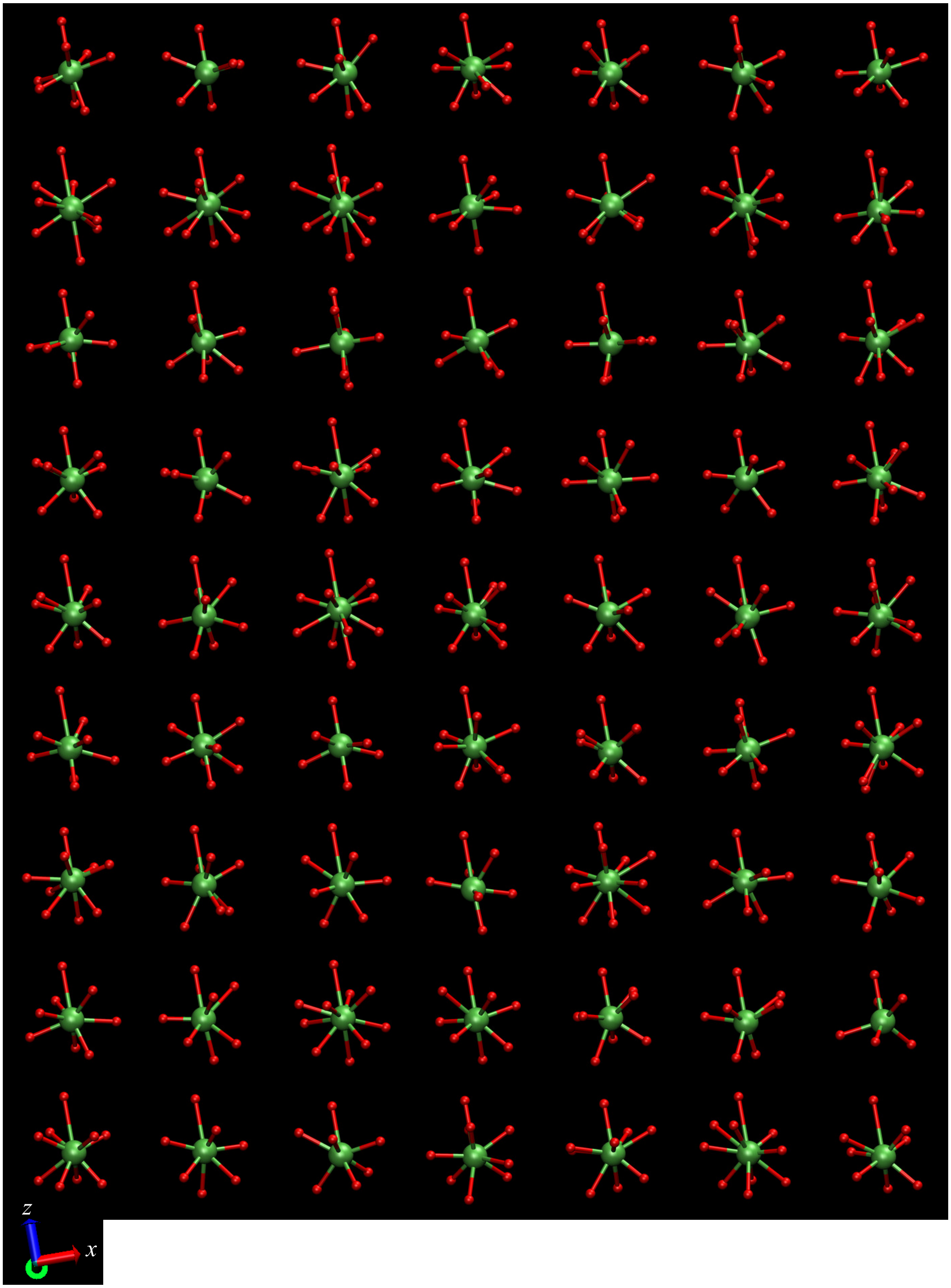

Fig. S9: All extracted La coordination structures for a-La60.
(continued on next page)

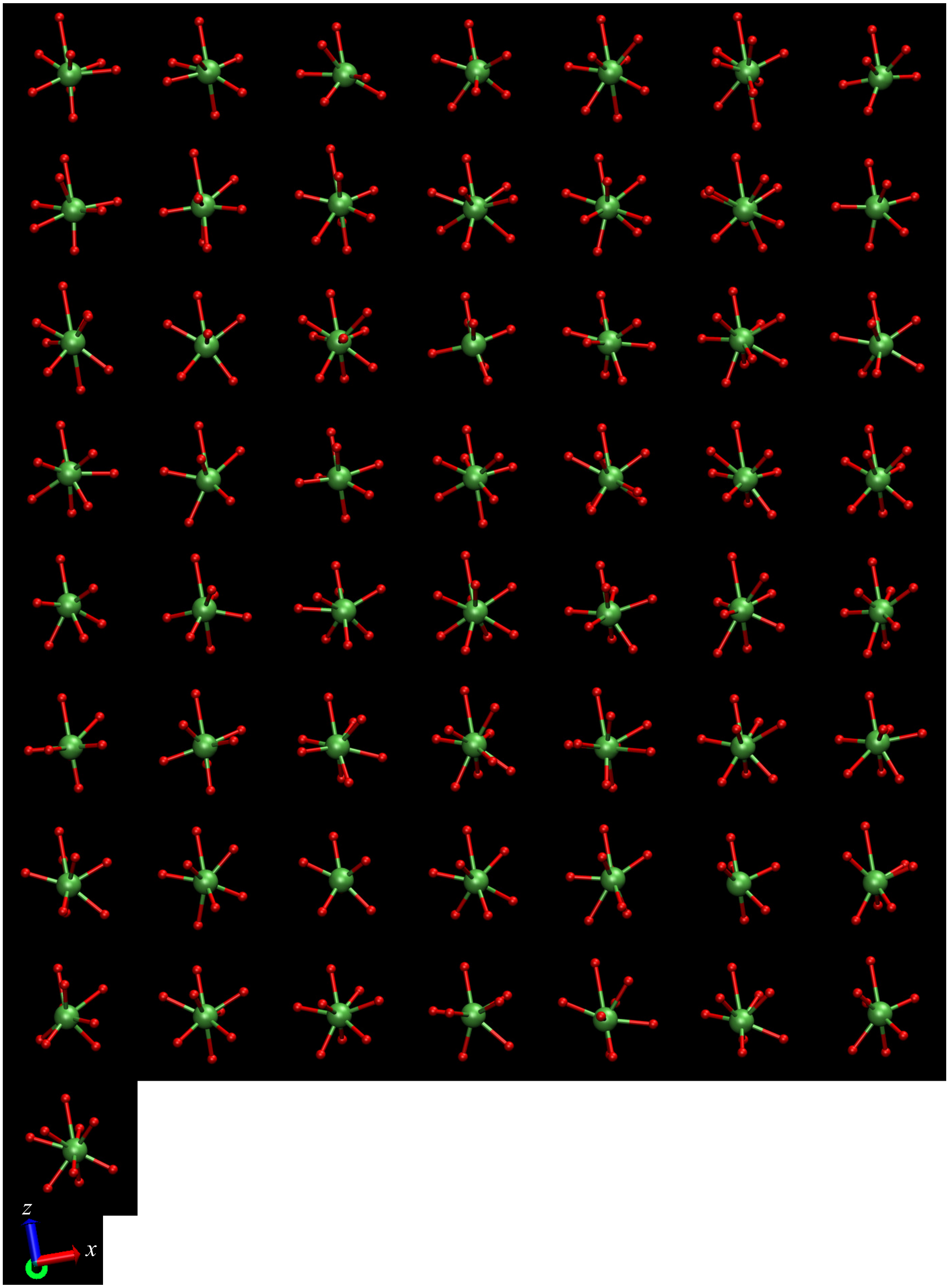

(Fig. S9 end)

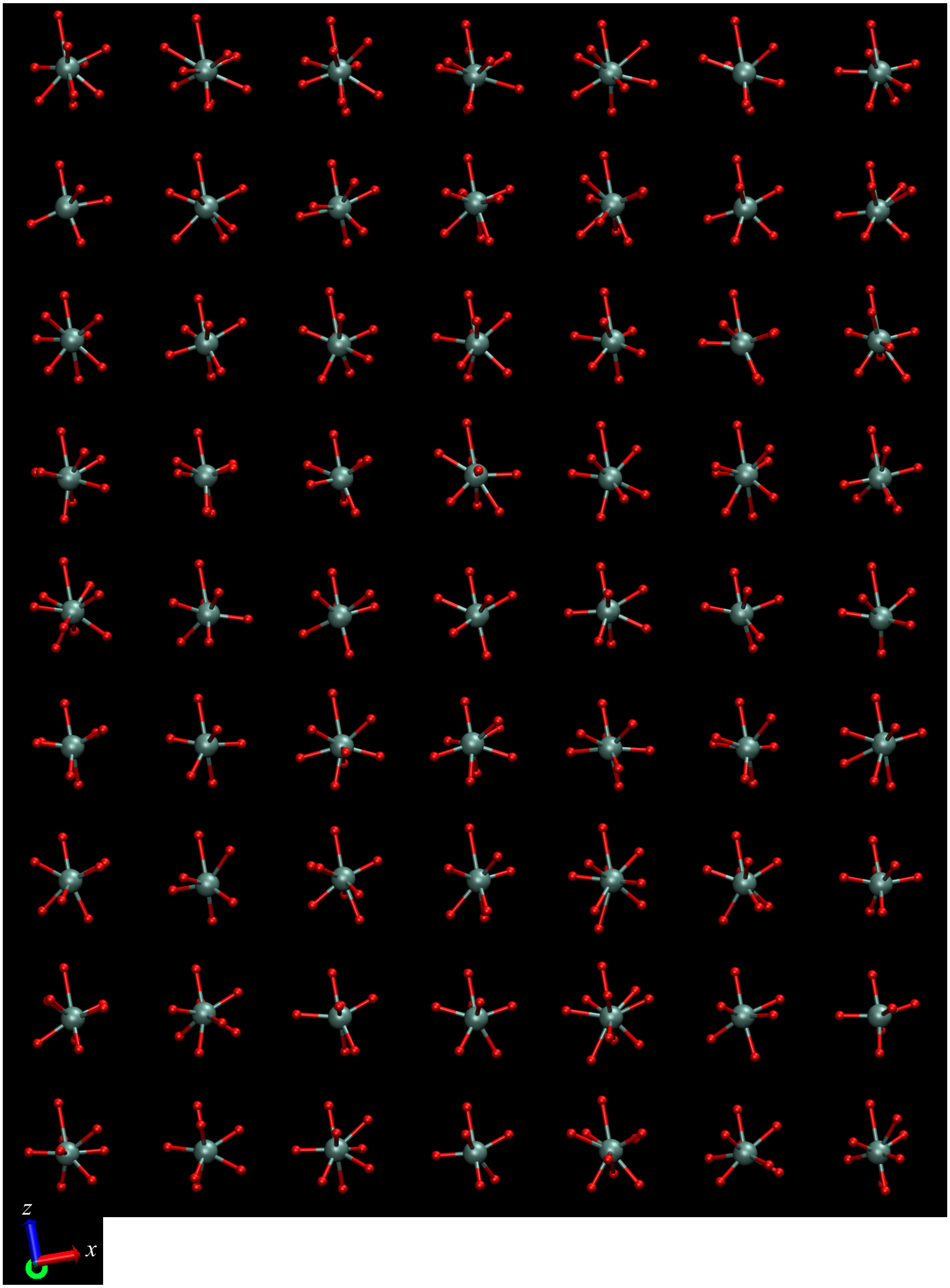

Fig. S10: All extracted Y coordination structures for a-Y50.
(continued on next page)

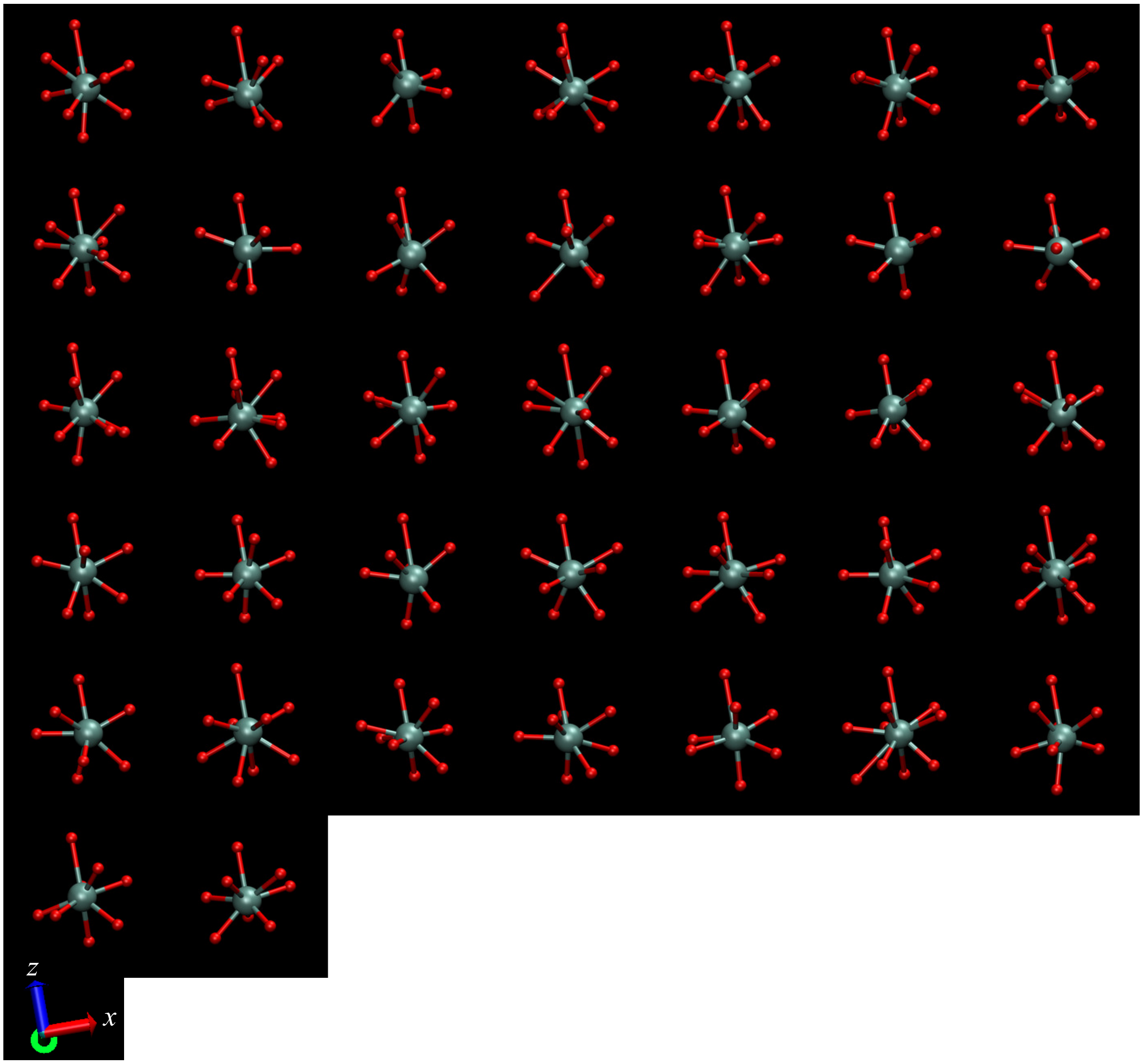

(Fig. S10 end)

The model structures derived from the solution of the Thomson problem[S5,S6] with CN = 6–12 are drawn in Fig. S11. The Thomson problem is an optimization problem in which point charges are placed on a sphere to achieve the smallest possible Coulomb potential. To facilitate comparison with the coordination structures extracted from the AIMD-derived glass structures, the center and point charges are depicted as La and O with 2.5 Å La–O distance. The parameters to represent the shape and symmetry of the model structures described in the main text are summarized in Table S3, along with the point groups of the model structures.

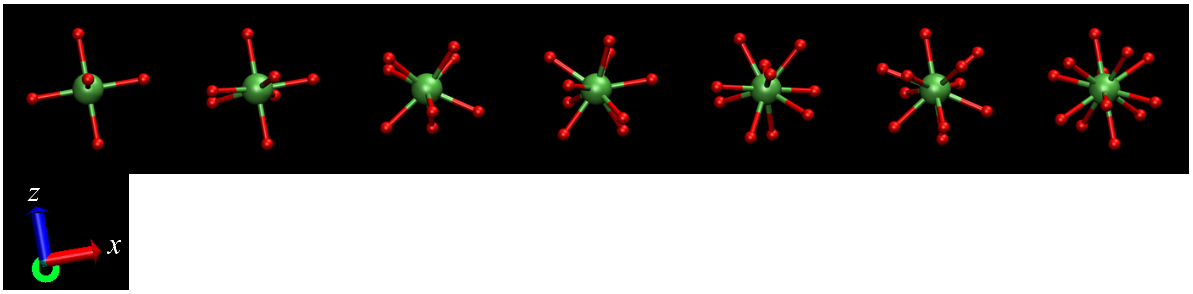

Fig. S11: Model coordination structures derived from the Thomson problem, with CN = 6–12 from left to right.

Table S3: Set of parameters to measure the reference structures derived from the solution of the Thomson problem. Here: P is the point group of the structure; I_{iso}, I_{ω}, and I_{κ} in Å are derived from the inertia tensor; the values of q_l ($l = 1$–6) are calculated from spherical harmonics; V_{vol} is the cell volume in Å^3; and V_3–V_7 are the indices of the Voronoi cells. Note that the parameters except I_{iso} and V_{vol} are invariant to the radius of the sphere.

CN	P	I_{iso}	I_{ω}	I_{κ}
6	O_h	5872.5	0.0	0.000
7	D_{5h}	7318.0	784.1	0.333
8	D_{4d}	8896.7	385.2	0.333
9	D_{3h}	10 608.8	77.6	0.333
10	D_{4d}	12 454.1	269.0	−0.333
11	C_{2v}	14 432.8	511.1	−0.136
12	I_h	16 544.9	0.0	−0.167

CN	q_1	q_2	q_3	q_4	q_5	q_6
6	0.000	0.000	0.000	0.764	0.000	0.354
7	0.000	0.071	0.000	0.554	0.501	0.063
8	0.000	0.029	0.000	0.371	0.585	0.254
9	0.000	0.005	0.074	0.148	0.607	0.343
10	0.000	0.014	0.000	0.076	0.506	0.451
11	0.000	0.021	0.043	0.107	0.344	0.543
12	0.000	0.000	0.000	0.000	0.000	0.663

CN	V_{vol}	V_3	V_4	V_5	V_6	V_7
6	20.833	0	6	0	0	0
7	24.767	0	5	2	0	0
8	27.343	0	8	0	0	0
9	31.869	0	3	6	0	0
10	34.572	0	2	8	0	0
11	36.735	0	2	8	1	0
12	39.627	0	0	12	0	0

Line profiles of electron density $\rho(x)$ along RE–O bonds are shown in Fig. S12 as a function of normalized distance $x/|r|$, where x is the distance from the RE and $|r|$ is the RE–O bond length.

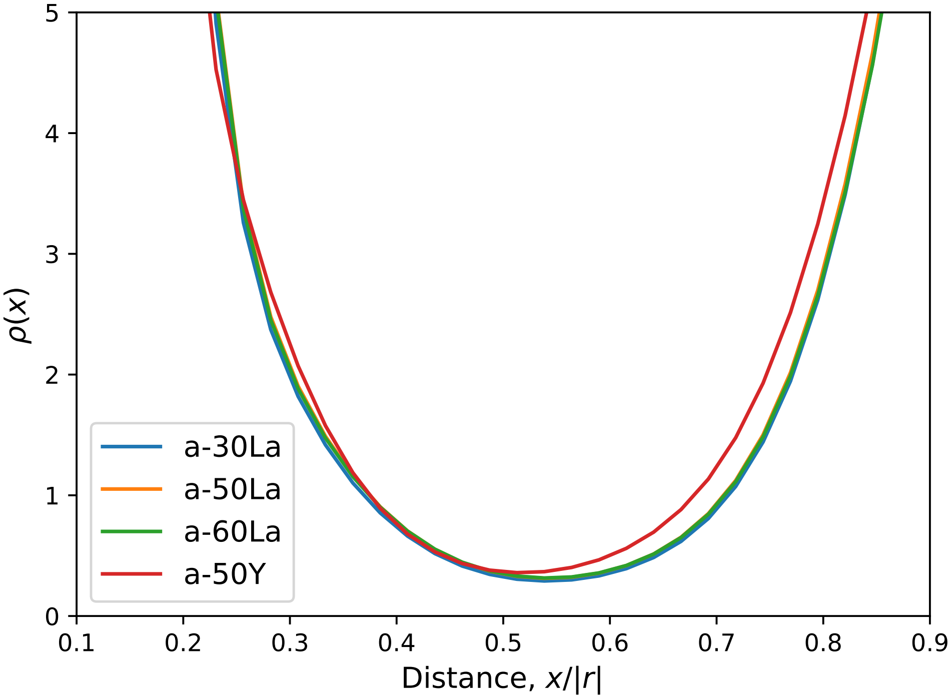

Fig. S12: Mean electron distributions along RE–O bonds for the a-La50, a-La30, a-La50, a-La60, and a-Y50 glasses. The positions x are normalized by the RE–O distance on each RE–O bond.

References

(S1) Mishra, K.; DeBoer, B.; Schmidt, P.; Osterloh, I.; Stephan, M.; Eyert, V.; Johnson, K. Electronic structures and nature of host excitation in borates. *Ber. Bunsen Ges. Phys. Chem.* **1998**, *102*, 1772–1782.

(S2) Abdullaev, G.; Dzhafarov, G.; Mamedov, K. S. Crystal structure of lanthanum orthoborate. *AZKZAU* **1976**, 117–120.

(S3) Momma, K.; Izumi, F. VESTA: a three-dimensional visualization system for electronic and structural analysis. *J. Appl. Crystallogr* **2008**, *41*, 653–658.

(S4) Sasaki, S.; Masuno, A.; Yanaba, Y.; Inoue, H.; Ohkubo, T. Survival of fragmentated BO4 units in highly modified rare-earth-rich borate glasses. *ChemRxiv* **2024**,

(S5) Erber, T.; Hockney, G. Equilibrium configurations of N equal charges on a sphere. *J. Phys. A: Math. Gen.* **1991**, *24*, L1369.

(S6) Edmundson, J. The distribution of point charges on the surface of a sphere. *Acta Crystallogr., Sect. A: Found. Crystallogr.* **1992**, *48*, 60–69.
